# The Germinal Center Kinase TNIK Is Required for Canonical NF-κB and JNK Signaling in B-Cells by the EBV Oncoprotein LMP1 and the CD40 Receptor

**DOI:** 10.1371/journal.pbio.1001376

**Published:** 2012-08-14

**Authors:** Anna Shkoda, Jennifer A. Town, Janine Griese, Michael Romio, Hakan Sarioglu, Thomas Knöfel, Fabian Giehler, Arnd Kieser

**Affiliations:** 1 Research Unit Gene Vectors, Helmholtz Zentrum München - German Research Center for Environmental Health, München, Germany; 2 Research Unit Protein Science, Helmholtz Zentrum München - German Research Center for Environmental Health, München, Germany; University of Wisconsin-Madison, United States of America

## Abstract

The tumor necrosis factor-receptor-associated factor 2 (TRAF2)- and Nck-interacting kinase (TNIK) is a ubiquitously expressed member of the germinal center kinase family. The TNIK functions in hematopoietic cells and the role of TNIK-TRAF interaction remain largely unknown. By functional proteomics we identified TNIK as interaction partner of the latent membrane protein 1 (LMP1) signalosome in primary human B-cells infected with the Epstein-Barr tumor virus (EBV). RNAi-mediated knockdown proved a critical role for TNIK in canonical NF-κB and c-Jun N-terminal kinase (JNK) activation by the major EBV oncoprotein LMP1 and its cellular counterpart, the B-cell co-stimulatory receptor CD40. Accordingly, TNIK is mandatory for proliferation and survival of EBV-transformed B-cells. TNIK forms an activation-induced complex with the critical signaling mediators TRAF6, TAK1/TAB2, and IKKβ, and mediates signalosome formation at LMP1. TNIK directly binds TRAF6, which bridges TNIK's interaction with the C-terminus of LMP1. Separate TNIK domains are involved in NF-κB and JNK signaling, the N-terminal TNIK kinase domain being essential for IKKβ/NF-κB and the C-terminus for JNK activation. We therefore suggest that TNIK orchestrates the bifurcation of both pathways at the level of the TRAF6-TAK1/TAB2-IKK complex. Our data establish TNIK as a novel key player in TRAF6-dependent JNK and NF-κB signaling and a transducer of activating and transforming signals in human B-cells.

## Abstract 2

TNIK has an important function in physiological activation and viral transformation of human B-cells by interacting with the TRAF6 adapter complex and mediating NF-κB and JNK signal transduction.

## Author Summary

The germinal center kinase family member TNIK was discovered in a yeast-two-hybrid screen for interaction partners of the adapter proteins TRAF2 and Nck, and here we show it is one of the missing molecular players in two key signaling pathways in B-lymphocytes. We found that TNIK is crucial for the activities of the CD40 receptor on Bcells and its viral mimic, the latent membrane protein 1 (LMP1) of Epstein-Barr virus (EBV). EBV is a human DNA tumor virus that is associated with various malignancies. It targets and transforms B-cells by hijacking the cellular signaling machinery via its oncogene LMP1. In normal Bcell physiology, the CD40 receptor is central to the immune response by mediating B-cell activation and proliferation. TNIK turns out to be an organizer of the LMP1- and CD40-induced signaling complexes by interacting with the TRAF6 adapter protein, well known for its role in linking distinct signaling pathways. Through this mechanism the two receptors depend on TNIK to activate the canonical NF-κB and JNK signal transduction pathways, which are important for the physiological activation of B-cells (a process that enables antibody production), as well as for their transformation into tumor cells. TNIK thus constitutes a key player in the transmission of physiological and pathological signals in human B-cells that might serve as a future therapeutic target against B-cell malignancies.

## Introduction

TNIK was discovered in a yeast-two-hybrid screen for interaction partners of the adapter proteins TRAF2 and Nck [1]. The serine/threonine kinase TNIK is a member of the germinal center kinase (GCK) family, which belongs to the Ste20 group of kinases [2]. GCKs share high sequence homology in their N-terminal kinase and C-terminal germinal center kinase homology (GCKH) domains, while the intermediate domain is less conserved [2]. Current knowledge about the molecular and biological functions of TNIK is very limited. TNIK overexpression modulates the actin cytoskeleton and activates the JNK pathway, which is induced through the GCKH domain by a yet undefined mechanism [1],[3]. The molecular function of TNIK's interaction with TRAF molecules is unclear. A recent study suggested that TRAF2 and TNIK might be located within one signaling pathway that leads to Wnt pathway induction in chronic myelogenous leukemia stem cells [4]. TNIK also mediates proliferative Wnt signals in crypts of the small intestine and colorectal cancer cells by nuclear translocation and subsequent phosphorylation of the transcription factor TCF4 [5],[6]. In neurons, TNIK is involved in the regulation of neurite growth and neuronal structure [7],[8]. However, a physiological role for TNIK in hematopoietic cells has not been described.

The latent membrane protein 1 (LMP1) of Epstein-Barr virus (EBV) serves as proto-type of a viral receptor-like oncoprotein that usurps cellular signal transduction pathways for cell transformation. The gamma-herpesvirus EBV, classified as a human DNA tumor virus by the WHO, establishes a chronic latent infection in B-cells and is associated with various malignancies, such as Hodgkin's and Burkitt's lymphoma, life-threatening post-transplant lymphoproliferative disorders, or nasopharyngeal carcinoma [9]. LMP1 is found expressed in most EBV-associated tumors and it is crucial for viral cell transformation and continued in vitro proliferation of latently EBV-infected B-cells, so-called lymphoblastoid cell lines (LCLs) [9]. LMP1 resembles a constitutively active cellular receptor whose ligand-independent signaling activity is attributable to spontaneous homo-oligomerization of LMP1 molecules within the membrane [10]. By the recruitment of TRAF molecules, LMP1 mimics molecular functions of the CD40 receptor in B-cell activation and proliferation. However, compared to CD40, LMP1 assembles a unique and more efficient signaling complex [11]–[14]. Constitutive expression of LMP1 in the B-cell compartment of transgenic mice induces lymphomas, whereas timely activation of LMP1 signaling largely substitutes for CD40 deficiency in B-cell development and function [15]–[17].

**Figure 1:**
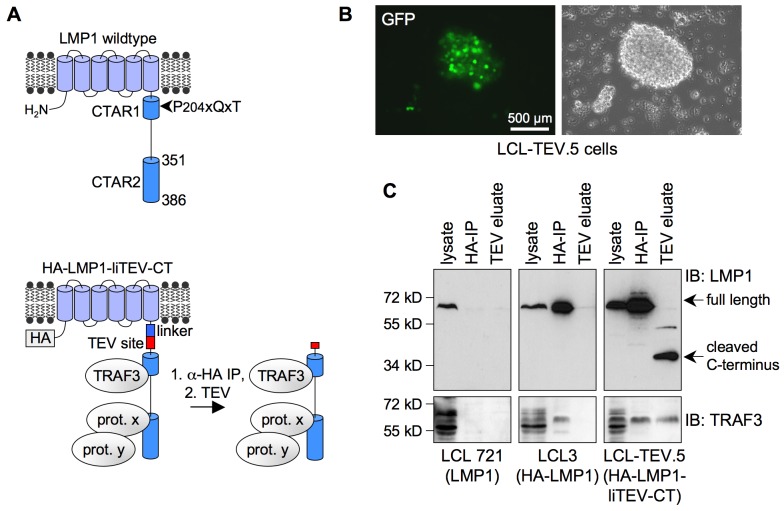
Experimental system for the proteomic identification of LMP1 interaction partners in EBV-infected primary human B-cells. (A) Schematic representation of the LMP1 wildtype molecule (top) and the engineered HA-LMP1-liTEV-CT derivative (bottom). For proteomics, HA-LMP1-liTEV-CT was immunoprecipitated by anti-HA-antibodies. Subsequently, the LMP1 signaling domain together with its interaction partners was cleaved off by TEV protease, eluted from the beads, and subsequently analyzed by immunoblotting or mass spectrometry. The known LMP1-binding protein TRAF3 and hypothetical direct and indirect interaction partners x and y, respectively, are shown as examples to illustrate the experimental approach. (B) Lymphoblastoid B-cell clone LCL-TEV.5 growing out from an infection of primary human B-cells with the recombinant maxi-EBV 2089-HA-LMP1-liTEV-CT, which has been generated by replacing the endogenous LMP1 gene of wildtype maxi-EBV 2089 by HA-LMP1-liTEV-CT. Expression of the GFP marker gene (left panel) in LCL-TEV.5 cells verified successful infection with recombinant virus. (C) Immunoprecipitation and TEV cleavage experiment. As indicated, the experiment was performed with LCLs expressing LMP1 wildtype (LCL 721, left panel), HA-LMP1 (LCL3, middle panel), or HA-LMP1-liTEV-CT (LCL-TEV.5, right panel). Anti-HA immunoprecipitations were performed (HA-IP, middle lanes). Precipitates were treated with TEV protease and the released LMP1 C-terminus together with bound proteins was eluted and analyzed by immunoblotting (TEV eluate, right lanes) or mass spectrometry (for the identification of TNIK in the TEV eluate of LCL-TEV.5 cells, see Tables S1 and S2). As expected, the tracer molecule TRAF3 was only found in the TEV eluate of LCL-TEV.5 but not of LCL 721 or LCL3 cells. The following antibodies were used for immunoblotting: anti-LMP1 (CS1-4) directed against the LMP1 signaling domain and anti-TRAF3 (C-20). Apparent molecular masses are given in kilodaltons (kDa). IB, immunoblot.

LMP1 consists of a short N-terminal domain (amino acids 1–24), six transmembrane helices, and a C-terminal cytoplasmic signaling domain (amino acids 187–386) (Figure 1A). The signaling domain harbors the two functionally distinct C-terminal activating regions (CTAR) 1 and 2, which activate the NF-κB, c-Jun N-terminal kinase (JNK), MAPK, PI3-kinase, and IRF7 signaling cascades [18]–[20]. The consensus TRAF binding motif P(204)xQxT is essential for CTAR1 function and directly binds TRAF1, 2, 3, and 5 [13],[21]–[23]. CTAR1 triggers the non-canonical NF-κB pathway involving IκB kinase α (IKKα)-induced processing of the NF-κB precursor p100 to p52 [24]–[27].

CTAR2 (amino acids 351–386) activates JNK and IκB-dependent canonical NF-κB, which contribute critical anti-apoptotic and proliferative signals for survival, proliferation, and tumorigenicity of EBV-transformed B-cells [25],. The TNFR1-associated death domain protein (TRADD) interacts with the 16 C-terminal amino acids of CTAR2 and is involved in NF-κB signaling by facilitating IKKβ recruitment to CTAR2 [34]–[36]. TRAF6 is essential for canonical NF-κB and JNK activation by CTAR2, although direct binding of TRAF6 to LMP1 has not been demonstrated [33],[37],[38]. Interaction of both proteins might thus be indirect involving the transcription factor BS69 as a mediator and/or stabilizer [39]. Downstream of TRAF6, the E2 ubiquitin-conjugating enzyme Ubc13, the TGFβ-receptor-associated kinase 1 (TAK1), and the TAK1-binding protein 2 (TAB2) as well as IKKβ and IKKγ play important roles in CTAR2 signaling [33],[37],[40]–[42].

Apart from LMP1, TRAF6 also mediates canonical NF-κB and JNK signaling by cellular receptors such as CD40 or Toll-like receptors [43]. Current concepts of TAK1 and IKKβ activation by TRAF6 have been reviewed [43]–[45]. In brief, activated and K63-autoubiquitinated TRAF6 binds TAB2, which then mediates the recruitment of the MAP3kinase TAK1 to TRAF6. TRAF6-derived unanchored ubiquitin chains bind TAB2 and help to induce TAK1 [46]. Activated TAK1 phosphorylates MKK6 to upregulate the JNK pathway. TAK1 also phosphorylates IKKβ within its activation loop and IKKβ activation is further facilitated by interaction of its regulatory component IKKγ with TRAF6. However, IKKβ is also induced by a TAK1-independent mechanism [44],[46]. IKKβ phosphorylates IκB, which results in IκB degradation and the release of active p65/p50 NF-κB dimers to the nucleus. It is tempting to speculate that yet unknown factors might serve as additional organizers or scaffolding proteins for TRAF-TAK-IKK complexes within the cell to orchestrate NF-κB and JNK signaling.

It is still not fully understood how the signaling complex at CTAR2 of LMP1 is assembled and how activation of transforming downstream signals is conveyed. We hypothesized the existence of still undefined molecular players and set out to identify novel LMP1 interaction partners by a functional proteomics approach. We report the characterization of TNIK as a component of the LMP1 signaling complex in EBV-transformed human B-cells. TNIK has a critical role in LMP1-induced JNK and canonical NF-κB signaling by the formation of an activation-induced complex at LMP1 with TRAF6, TAK1/TAB2, and IKKβ. Accordingly, TNIK is required for proliferation and survival of lymphoblastoid cells. TNIK is also of critical importance for physiological activation of the two pathways in B-cells by the CD40 receptor. Taken together, we identified TNIK as a novel key player in TRAF6-dependent JNK and NF-κB activation by two members of the TNF receptor family.

## Results

### Functional Proteomics Identifies TNIK as LMP1 Interaction Partner

We set out to identify novel interaction partners of the LMP1 signaling complex in its native context, the EBV-transformed primary human B-cell. To this end, HA-LMP1-liTEV-CT, an LMP1 variant optimized for proteomics studies, was expressed from a recombinant maxi-EBV genome in lymphoblastoid cells. To generate HA-LMP1-liTEV-CT, an N-terminal hemagglutinin (HA)-tag was added and a tobacco etch virus protease cleavage site coupled to a flexible linker (liTEV) was inserted between the transmembrane domain and the C-terminal (CT) signaling domain of LMP1 (Figure 1A). TEV protease cleavage after immunoprecipitation of the HA-LMP1-liTEV-CT complex allowed the release of the LMP1 signaling domain and its interaction partners from the beads for further analysis by mass spectrometry. By this approach the background of proteins was reduced which either interacted with the LMP1 N-terminus and/or the transmembrane domain or which unspecifically bound to the beads or antibodies.

Recombinant EBV expressing HA-LMP1-liTEV-CT from the viral LMP1 promoter was used to infect primary B-cells isolated from human adenoids. The recombinant virus efficiently transformed B-cells into lymphoblastoid cells, which showed typical clumpy LCL growth and green fluorescence due to the expression of a green fluorescence protein (GFP) marker gene located on the recombinant virus genome (Figure 1B). The clone LCL-TEV.5 was used for proteomics studies. The outgrowth of LCL-TEV cells further proved that HA-LMP1-liTEV-CT was fully functional because an intact LMP1 is mandatory for B-cell transformation by EBV [11]. Moreover, HA-LMP1-liTEV-CT was able to induce signaling as wildtype LMP1 in HEK293 cells (Figure S1).

HA-LMP1-liTEV-CT was immunoprecipitated from lysates of LCL-TEV.5 cells (Figure 1C). Parallel precipitations were performed with the lymphoblastoid cell lines LCL 721 expressing wildtype LMP1 and LCL3 expressing HA-tagged LMP1 [35]. Expression levels of the LMP1 proteins were comparable in all three cell types (Figure 1C). HA-LMP1-liTEV-CT and HA-LMP1 were efficiently immunoprecipitated by anti-HA antibodies. TEV protease cleavage released the signaling domain of HA-LMP1-liTEV-CT but not that of HA-LMP1 (Figure 1C). The known CTAR1 interaction partner TRAF3 verified functionality of the experimental system. As expected, TRAF3 specifically co-precipitated with both HA-tagged LMP1 variants but was only detected in the TEV eluate of LCL-TEV.5 immunoprecipitations (Figure 1C). TEV eluates of LCL-TEV.5 immunoprecipitations were analyzed by mass spectrometry as described in Materials and Methods. The identified candidate LMP1 interaction partners included signaling proteins, proteins involved in ubiquitinylation processes, cytoskeletal proteins, and proteins with other or unknown functions. Two peptides identifying the TRAF2- and Nck-interacting kinase (TNIK) were detected in the TEV eluate of LCL-TEV.5, but not of control cells, which indicated that TNIK interacts with the signaling domain of HA-LMP1-liTEV-CT and is thus a novel component of the LMP1 signaling complex (Tables S1 and S2).

### LMP1 Recruits TNIK via the CTAR2 Domain

**Figure 2:**
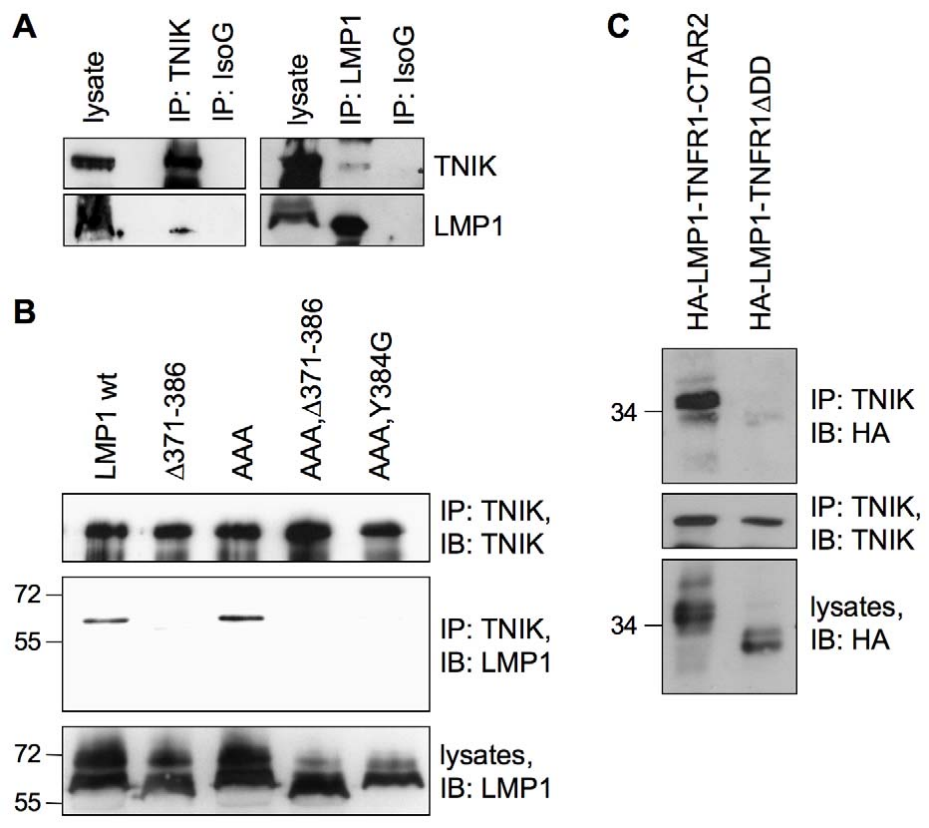
TNIK binds to the LMP1 signaling complex. (A) Endogenous TNIK interacts with LMP1 in lymphoblastoid cells. TNIK was immunoprecipitated from LCL 721 cell lysates with the anti-TNIK antibody (left panel). Co-precipitated wildtype LMP1 was detected by the anti-LMP1 (1G6-3) antibody. An unrelated mouse isotype IgG (IsoG) served as a negative control for immunoprecipitation. Vice versa, endogenous LMP1 was immunoprecipitated using the anti-LMP1 (1G6-3) antibody (right panel). Co-precipitated TNIK was detected using the anti-TNIK antibody. Rat isotype IgG served as a negative control. IB, immunoblot; IP, immunoprecipitation. Shown is one representative of four independent experiments (*n* = 4). (B) The CTAR2 domain of LMP1 is essential for TNIK recruitment. HEK293 cells were transfected in 15 cm culture dishes with 20 µg of the indicated LMP1 constructs. The Δ371-386 deletion inactivates the CTAR2 domain. CTAR2 with Y384G mutation has no signaling capacity towards NF-κB and JNK. AAA harbors a mutated TRAF interaction motif within CTAR1. Endogenous TNIK was immunoprecipitated from cell lysates. LMP1 co-precipitation was analyzed using the anti-LMP1 (CS1-4) antibody. The anti-TNIK antibody was used to verify equal TNIK precipitation in all samples. Comparable expression of LMP1 constructs was confirmed in cell lysates. *n* = 3. (C) The 16 C-terminal amino acids of CTAR2 are sufficient for interaction with TNIK. HEK293 cells were transfected with pCMV-HA-LMP1-TNFR1ΔDD, a chimera of the LMP1 transmembrane domain and the signaling domain of TNFR1 lacking its death domain (DD), or pCMV-HA-LMP1-TNFR1-CTAR2 carrying amino acids 371–386 of LMP1 instead of DD. Endogenous TNIK was immunoprecipitated from cell lysates and co-precipitation of the HA-tagged chimeras was analyzed by immunoblotting with the anti-HA (3F10) antibody. The anti-TNIK antibody was used to confirm comparable TNIK precipitation. *n* = 3.

To confirm the interaction between TNIK and LMP1 in lymhoblastoid cells, endogenous TNIK was immunoprecipitated from lysates of LCL 721 cells and analyzed for LMP1 binding (Figure 2A). Indeed, endogenous LMP1 specifically co-precipitated with TNIK. Vice versa, immunoprecipitation of LMP1 brought down TNIK (Figure 2A). These experiments verified the results that were previously obtained in the functional proteomics experiment and showed that TNIK is in fact part of the LMP1 signalosome in EBV-transformed B-cells.

Next we asked whether one of the two signaling-active subdomains of LMP1, CTAR1 or CTAR2, mediates the interaction between TNIK and LMP1. Wildtype LMP1 as well as LMP1(AAA) harboring a mutated PxQxT motif within CTAR1, the CTAR2 deletion mutant LMP1Δ371–386, and the CTAR1/CTAR2 double mutants LMP1(AAA, Δ371–386) and LMP1(AAA, Y384G) were transiently expressed in HEK293 cells and endogenous TNIK was immunoprecipitated from cell lysates. Immunoblot analysis of the precipitations revealed that wildtype LMP1 and the LMP1(AAA) mutant bound to TNIK equally well, excluding a critical role of CTAR1 for TNIK binding. In contrast, mutation of CTAR2 completely abolished interaction of LMP1 and TNIK, the exchange of tyrosine 384 to glycine being equally effective as the deletion of the 16 C-terminal amino acids of CTAR2 (Figure 2B). These experiments indicated but did not definitely prove that CTAR2 is the critical domain for LMP1's interaction with TNIK. Therefore, we repeated the experiment with the HA-LMP1-TNFR1-CTAR2 chimera, which consists of the LMP1 transmembrane domain fused to the TNFR1 signaling domain that carries amino acids 371 to 386 of CTAR2 replacing the TNFR1 death domain [35]. Except for CTAR2 residues 371–386, no other sequences of the LMP1 signaling domain are present within the chimera. TNIK readily bound to HA-LMP1-TNFR1-CTAR2 but not the control construct lacking the CTAR2 sequences (Figure 2C). In summary, these experiments demonstrated that CTAR2 is both critical and sufficient for TNIK recruitment to LMP1, whereas CTAR1 has no apparent role in mediating this interaction.

### TNIK Is Essential for JNK Activation by LMP1

**Figure 3:**
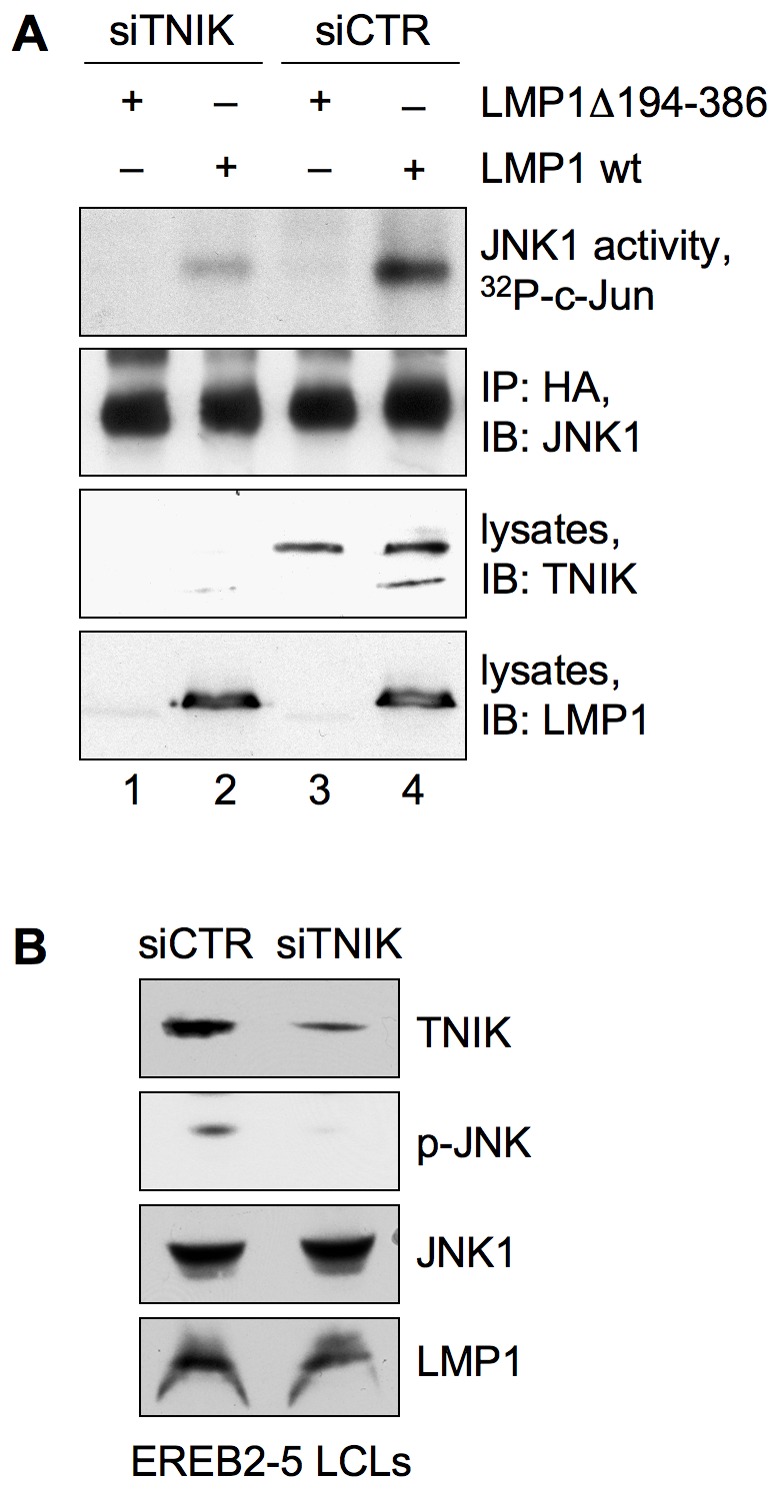
TNIK is essential for JNK activation by LMP1. (A) HEK293 cells were transfected in 6-well plates with siRNA against human TNIK (siTNIK) or non-targeting control siRNA (siCTR). Subsequently, the cells were co-transfected with 1 µg of HA-JNK1 and 1 µg of HA-LMP1 wildtype or HA-LMP1Δ194–386 lacking the LMP1 signaling domain, as indicated. HA-JNK1 was immunoprecipitated from cell lysates and immunocomplex kinase assays were performed using recombinant GST-c-Jun as a substrate. Equal HA-JNK1 immunoprecipitation was confirmed by the anti-JNK1 antibody. TNIK downregulation and LMP1 expression was monitored in cell lysates using anti-TNIK and anti-LMP1 (CS1-4) antibodies. The null control construct LMP1Δ194–386 cannot be detected by the CS1-4 antibody because all CS1-4 epitopes are located within the LMP1 signaling domain. Quantification of four independent experiments ± standard deviations, given as x-fold inductions: lane 1, 1.15±0.32; lane 2, 1.48±0.6; lane 3, 1.0±0.0; lane 4, 3.23±1.03. (B) The knockdown of TNIK in lymphoblastoid cells blocks the JNK pathway. EREB2-5 B-cells were treated with Accell siRNA against human TNIK or non-targeting siRNA for 72 h. Cell lysates were analyzed by immunoblotting using anti-TNIK, anti-phospho-JNK, anti-JNK1, and anti-LMP1 (CS1-4) antibodies. *n* = 3.

Having identified TNIK as a novel CTAR2 interaction partner, we asked whether TNIK has a role in LMP1 signal transduction. The JNK pathway initiates at CTAR2 and TNIK was shown to induce JNK signaling upon overexpression [1],[30]. Therefore, we investigated a potential role for TNIK as mediator of LMP1-induced JNK signal transduction. HEK293 cells were transfected with TNIK-specific siRNA or non-targeting control siRNA. Subsequently, wildtype LMP1 or the null control LMP1Δ194–386 were expressed, and HA-JNK kinase assays were performed to monitor LMP1-induced JNK1 activity. The knockdown of TNIK caused a drastic reduction of JNK activation by LMP1 (Figure 3A). We confirmed this result in the human lymphoblastoid cell line EREB2-5. Upon knockdown of TNIK with siRNA a robust reduction of endogenous JNK phosphorylation, a measure of JNK activity, was detected in EREB2-5 cells (Figure 3B). Notably, JNK activity in LCLs depends on LMP1 [11],[30],[47]. We have thus identified TNIK as a novel signaling mediator of LMP1 that is crucial for the induction of the JNK pathway.

### LMP1-Induced Canonical NF-κB Signaling Requires TNIK

**Figure 4:**
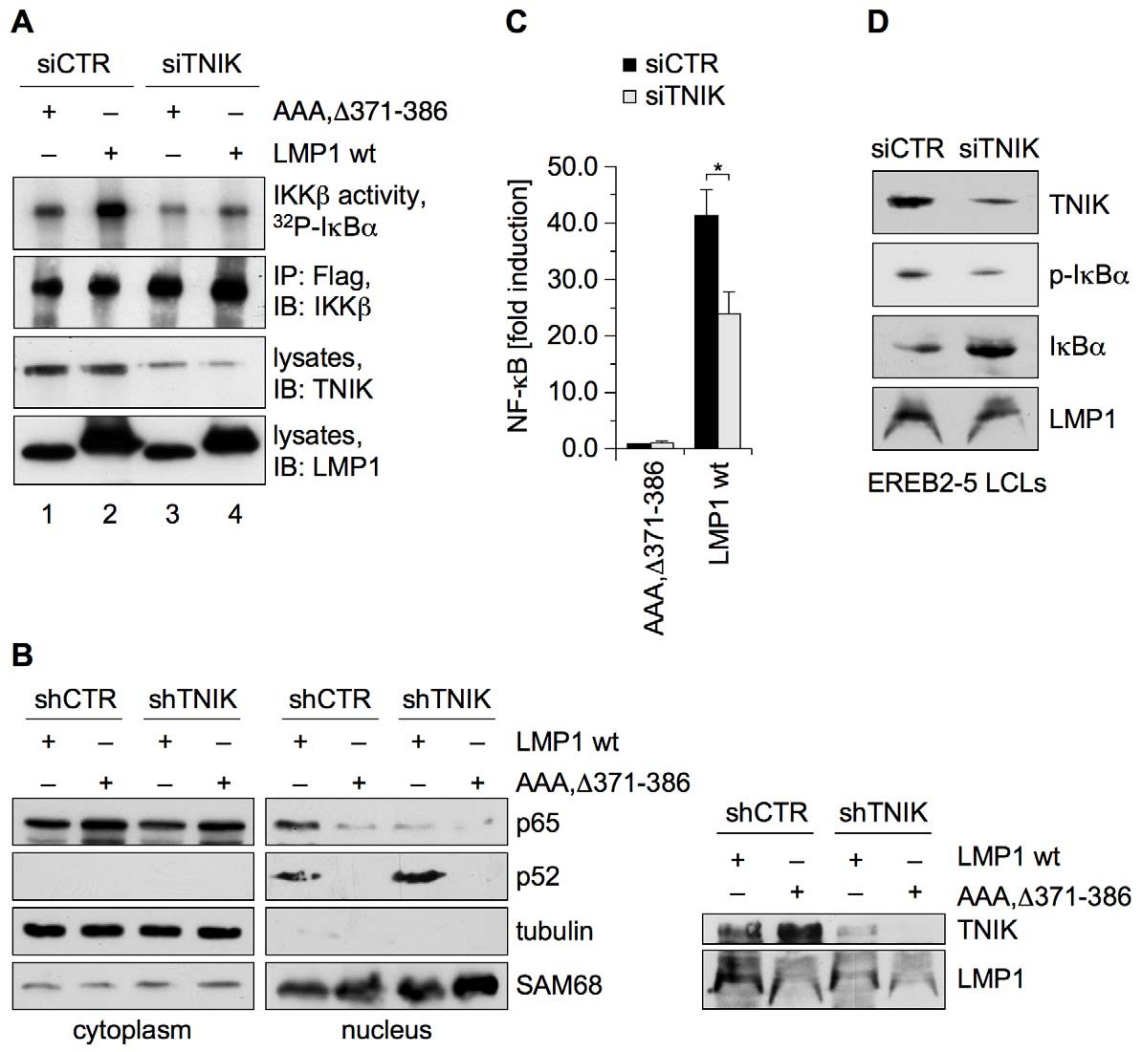
TNIK has a critical role in LMP1-induced canonical NF-κB signaling. (A) The knockdown of TNIK inhibits LMP1 activation of IKKβ. HEK293 cells were transfected in 6-well plates with TNIK siRNA or non-targeting siRNA. Subsequently, the cells were co-transfected with 1 µg each of HA-LMP1 wildtype or inactive HA-LMP1(AAA, Δ371–386) together with 1 µg of Flag-IKKβ, as indicated. Flag-IKKβ was immunoprecipitated and its activity was monitored in in vitro kinase assays using purified GST-IκBα as a substrate. Immunoprecipitated Flag-IKKβ, expression of LMP1 constructs, and downregulation of TNIK were detected in lysates using the anti-IKKβ, anti-LMP1 (1G6-3), and anti-TNIK antibodies, respectively. Quantification of three independent experiments: lane 1, 1.0±0.0; lane 2, 2.6±0.17; lane 3, 0.5±0.0; lane 4, 0.97±0.47. (B) TNIK mediates LMP1-induced canonical NF-κB signaling without affecting the non-canonical pathway. HEK293 cells were transfected in 10 cm culture dishes with 4 µg each of HA-LMP1 wildtype or HA-LMP1(AAA, Δ371–386). As indicated, 4 µg of pSM2-shTNIK expressing a short hairpin RNA targeting TNIK or non-targeting vector were co-transfected. 48 h post-transfection the cells were lysed, and cytoplasmic and nuclear fractions were prepared and analyzed by immunoblotting using the indicated antibodies for the NF-κB proteins p65 and p52. Tubulin and SAM68 were used as markers for the cytoplasmic and nuclear fractions, respectively. TNIK knockdown and LMP1 expression was monitored in total cell lysates (right). *n* = 2. (C) NF-κB reporter assays in HEK293 cells. TNIK was depleted from HEK293 cells by transfection in 6-well plates with TNIK siRNA. Cells were co-transfected with 2 µg of expression vectors for HA-LMP1(AAA, Δ371–386) or HA-LMP1 wildtype together with 5 ng of the NF-κB reporter 3xκBLuc and Renilla control vector. 24 h post-transfection cells were lysed and NF-κB reporter assays were performed. Luciferase activities were corrected for transfection efficiencies as described in Materials and Methods. Given data are mean values of three independent experiments ± standard deviations; statistics, two-tailed Student's *t* test. *p = 0.006. (D) The downregulation of TNIK in lymphoblastoid cells impairs the canonical NF-κB pathway. Identical samples of siTNIK or siCTR-treated EREB2-5 LCLs as shown in Figure 3B were blotted for phospho-IκBα and IκBα. Control immunoblots for TNIK and LMP1 expression are displayed again for better comparison. *n* = 3.

Canonical NF-κB constitutes the second important signaling pathway that is initiated at the CTAR2 domain of LMP1. CTAR2, but not CTAR1, induces IKKβ activity, which is essential for CTAR2-mediated NF-κB signaling [25],[35],[42]. To test if TNIK is involved in this pathway as well, Flag-IKKβ kinase assays were performed in HEK293 cells (Figure 4A). Endogenous TNIK was depleted by TNIK siRNA, and LMP1 wildtype or the inactive null mutant LMP1(AAA, Δ371–386) was expressed and tested for their ability to activate IKKβ. LMP1 expression in cells treated with control siRNA caused a 2.6-fold induction of IKKβ activity, monitored as in vitro GST-IκBα substrate phosphorylation by the immunoprecipitated Flag-IKKβ. The knockdown of TNIK almost entirely abolished the activation of IKKβ by LMP1, demonstrating the critical importance of TNIK in the canonical NF-κB pathway (Figure 4A). In order to exclude a role for TNIK in non-canonical NF-κB signaling by LMP1, the effect of a TNIK knockdown on NF-κB p52 was examined. NF-κB p52 activation is a hallmark for CTAR1 signaling [24]–[27]. Downregulation of TNIK by a shRNA vector in HEK293 cells did not affect the LMP1-induced p52 translocation to the nucleus, whereas the nuclear shift of canonical p65 was largely inhibited (Figure 4B).

NF-κB reporter assays were performed in HEK293 cells to test the role of TNIK also at the level of NF-κB-dependent transcription. The siRNA-mediated knockdown of TNIK caused a nearly 50% reduction in NF-κB activation by LMP1 as compared to cells treated with control siRNA (Figure 4C). Given that a substantial proportion of total LMP1-induced NF-κB activity detected in reporter assays constitutes CTAR1-induced non-canonical NF-κB [28],[29], we concluded that knockdown of TNIK largely blocked CTAR2 signaling in the reporter assay. This conclusion was later corroborated by the use of a dominant-negative TNIK mutant that inhibited CTAR2, but not CTAR1, activation of the NF-κB reporter (see Figure 6F).

We confirmed our findings by siRNA experiments in EBV-transformed EREB2-5 cells. Knockdown of TNIK in these cells resulted in a marked reduction of phosphorylated IκBα and a concomitant stabilization of IκBα showing that canonical NF-κB signaling is defective upon depletion of TNIK in LCLs (Figure 4D). TNIK is thus an important signaling mediator of the canonical NF-κB pathway.

### TNIK Mediates Proliferation and Survival of EBV-Transformed B-Cells

**Figure 5:**
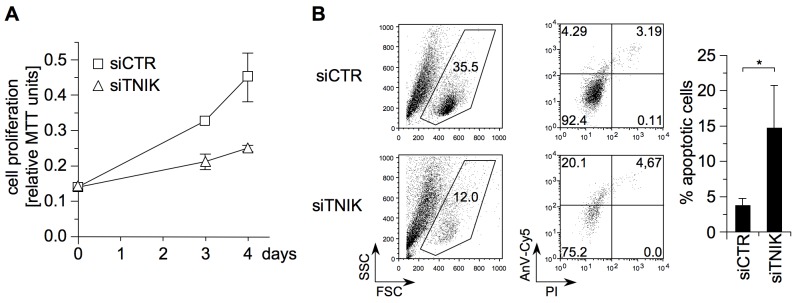
TNIK mediates proliferation and survival in EBV-transformed human B-cells. (A) The knockdown of TNIK inhibits proliferation of LCLs. EREB2-5 cells were seeded in the presence of Accell siRNA targeting TNIK or non-targeting siRNA. Cell proliferation was monitored at the indicated times by MTT conversion. Shown are the results of one representative experiment in triplicates of three independent experiments. (B) Apoptosis induction by TNIK knockdown in LCLs. EREB2-5 cells were incubated with Accell siTNIK or siCTR, as indicated. Apoptosis was monitored after 3 d in the presence of siRNA by co-staining of the cells with Cy5-labeled annexin V (AnV-Cy5) and propidium iodide (PI). The population of intact cells within the lymphocyte gate in the forward scatter (FSC)/sideward scatter (SSC) plot (left panels) was strongly reduced after the knockdown of TNIK. The gated cells were then analyzed for apoptosis rates, indicated by PI−/AnV-Cy5+ staining (right panels). The graph shows mean values of PI−/AnV-Cy5+ percentages of three independent experiments ± standard deviations; two-tailed Student's *t* test. *p = 0.033.

The LMP1-induced IκB-dependent NF-κB pathway and the JNK pathway are essential for lymphoblastoid cell survival and proliferation [31],[32]. Given the important role of TNIK in both pathways, its knockdown should interfere with LCL physiology. To test this hypothesis, TNIK expression was downregulated in EREB2-5 lymphoblastoid cells by siRNA and proliferation was monitored. In fact, TNIK deficiency strongly retarded proliferation of the cells and apoptosis was induced concomitantly (Figure 5A and 5B, respectively). The spontaneous apoptosis rate in EREB2-5 cells increased by a factor of 3.8 on average after the knockdown of TNIK (Figure 5B). Accordingly, many dead cells were visible in disintegrating LCL clumps in the siTNIK-treated EREB2-5 culture, whereas the siCTR-treated cells displayed normal LCL morphology (Figure S2). Thus, TNIK has a critical function in mediating proliferation and survival of LCLs, which is in line with its central role in LMP1 signal transduction.

### The JNK and Canonical NF-κB Pathways Bifurcate at the Level of TNIK

**Figure 6:**
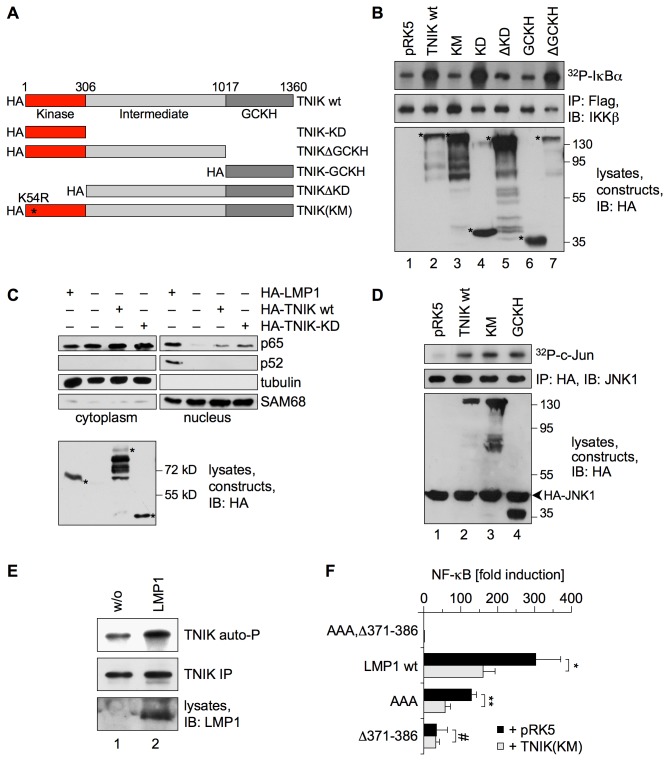
The JNK and canonical NF-κB pathways bifurcate at the level of TNIK. (A) Schematic diagram of TNIK mutants cloned with an N-terminal HA-tag. Amino acid positions are given in numbers. The asterisk marks the position of the K54R mutation that inactivates the kinase. GCKH, germinal center kinase homology domain; KD, kinase domain; KM, kinase mutant. (B) The intact TNIK kinase domain is required for IKKβ activation. HEK293 cells were transfected in 6-well plates with 2 µg of the indicated TNIK constructs or pRK5 empty vector and 1 µg of Flag-IKKβ. Flag-IKKβ activity was monitored in immunocomplex kinase assays. Immunoprecipitated Flag-IKKβ and expressed TNIK-constructs were detected using the anti-IKKβ and anti-HA (3F10) antibodies, respectively. Asterisks indicate the TNIK constructs. Apparent molecular masses are given in kDa. Quantification of three independent experiments: lane 1, 1.0±0.0; lane 2, 4.73±1.0; lane 3, 0.93±0.46; lane 4, 11.0±5.86; lane 5, 1.3±0.56; lane 6, 1.4±0.62; lane 7, 5.13±1.72. (C) The TNIK kinase domain induces the nuclear shift of canonical p65 NF-κB but not of non-canonical p52 NF-κB. HEK293 cells were transfected in 10 cm dishes with 4 µg each of the indicated constructs. LMP1 served as positive control. Cytoplasmic and nuclear fractions were prepared and analyzed by immunoblotting using the indicated antibodies. Expression of the constructs was verified in total lysates by the anti-HA (3F10) antibody. *n* = 4. (D) The GCKH domain of TNIK is sufficient for JNK activation and TNIK kinase activity is dispensable. HEK293 cells were transfected with 0.5 µg of the indicated HA-tagged TNIK constructs or pRK5 empty vector and 1 µg of HA-JNK1. Subsequently, immunocomplex kinase assays were performed. Immunoprecipitated HA-JNK1 and the expressed TNIK-constructs were detected using the anti-JNK1 and anti-HA (3F10) antibodies. Quantification of three independent experiments: lane 1, 1.0±0.0; lane 2, 3.2±1.66; lane 3, 4.57±1.35; lane 4, 6.0±0.1. (E) LMP1 enhances autophosphorylation of TNIK. HEK293 cells were transfected in 6-well plates with 1 µg of HA-TNIK vector together with or without 1 µg of pSV-LMP1, as indicated. Subsequently, HA-TNIK was immunoprecipitated from cell lysates using the anti-HA (12CA5) antibody and TNIK autophosphorylation reaction was performed with the precipitated TNIK in the presence of radioactive ATP. Equal TNIK precipitation and LMP1 expression was confirmed on immunoblots. Quantification of six independent experiments: lane 1, 1.0±0.0; lane 2, 4.28±2.76; two-tailed Student's *t* test, *p* = 0.015. (F) Kinase-negative TNIK(KM) exerts a dominant-negative effect specifically on the NF-κB pathway induced by CTAR2. HEK293 cells were transfected in 6-well plates with 1 µg of the indicated LMP1 constructs, 2 µg of kinase-negative HA-TNIK(KM) or empty vector, and 5 ng of the NF-κB reporter 3xκBLuc and Renilla control reporter. Luciferase activities were corrected for transfection efficiencies. Given data are mean values of three independent experiments ± standard deviations; two-tailed Student's *t* test. *p = 0.03, **p = 0.003, ^#^ *p* = 0.88.

As TNIK is critically involved in both JNK and canonical NF-κB signal transduction downstream of LMP1, we next asked whether these two pathways might bifurcate at the level of TNIK by dissecting the contribution of individual TNIK domains to the activation of JNK and NF-κB signaling. A set of HA-tagged TNIK constructs was generated that comprise full-length TNIK, the kinase domain (KD), the germinal center kinase homology domain (GCKH), as well as the ΔKD and ΔGCKH deletion mutants (Figure 6A). Additionally, a kinase-negative mutant (KM) of TNIK was used, which carries a mutation of the conserved lysine 54 residue in the ATP-binding pocket of the kinase domain [1]. We then tested for the ability of the individual TNIK constructs to induce canonical NF-κB signaling in IKKβ kinase activity assays. Wildtype TNIK activated IKKβ-dependent phosphorylation of GST-IκBα by a factor of 4.7-fold (Figure 6B). Notably, expression of the TNIK kinase domain alone was sufficient to fully induce IKKβ as TNIK-KD caused an 11-fold activation of IKKβ. Vice versa, mutation or deletion of the kinase domain completely abolished TNIK's potential to activate IKKβ. In contrast, neither deletion of the GCKH domain nor its overexpression had any effect on IKKβ activation. In line with these results, the exogenous expression of TNIK wildtype or TNIK-KD was sufficient to also induce the nuclear translocation of canonical NF-κB p65, whereas non-canonical NF-κB p52 remained unaffected (Figure 6C). This finding further corroborated our previous observations that TNIK has no function in non-canonical NF-κB signaling (see above). As expected, TNIK-KM was unable to shift any of the two NF-κB proteins to the nucleus (unpublished data). In summary, we concluded that the TNIK kinase domain and in particular its kinase activity is critical for canonical NF-κB induction by TNIK, while the GCKH domain is dispensable for this pathway.

Notably, JNK activation maps to a region of TNIK different from the NF-κB-activating kinase domain. The GCKH domain alone activates JNK as efficiently as full-length TNIK, whereas mutation of the kinase domain had no effect on TNIK's ability to induce JNK as determined by kinase assays in HEK293 cells (Figure 6D). This finding is consistent with previous results showing that the GCKH domain alone can induce the JNK pathway whereas the TNIK kinase domain is dispensable [1]. Taken together, JNK and IKKβ induction map to different TNIK domains, suggesting that TNIK constitutes the point of bifurcation of these two pathways.

Next we asked about the functional role of the TNIK kinase domain in IKKβ/NF-κB activation. One straightforward scenario would be that TNIK phosphorylates IKKβ for its activation. However, we did not detect direct IKKβ phosphorylation by TNIK in our experimental systems, for instance in TNIK kinase assays using IKKβ as a substrate (unpublished data). Previous studies demonstrated that TNIK phosphorylates itself [1],[3]. Therefore, we investigated if LMP1 expression affects TNIK autophosphorylation. In fact, LMP1 enhanced the phosphorylating activity of TNIK versus itself by a factor of 4.2-fold, demonstrating a role for TNIK autophosphorylation in LMP1 signaling (Figure 6E). The vast majority of the about 40 Ser/Thr phosphorylation sites of TNIK detected so far in vivo by mass spectrometry are located within the intermediate domain (databank: www.phosphosite.org; search term: TNIK). If the TNIK kinase domain phosphorylates TNIK within its intermediate domain and TNIK autophosphorylation is critical for NF-κB signaling, the exogenously expressed TNIK kinase domain alone would be non-functional but depend on endogenous wildtype TNIK to activate IKKβ. To test this possibility, HEK293 cells were depleted of endogenous wildtype TNIK by siRNA. Subsequently, the construct HA-TNIK-KDwob was transfected, which expresses the wildtype TNIK kinase domain, and IKKβ kinase assays were performed. As the HA-TNIK-KDwob construct carries silent wobble mutations at the nucleotide level, it is not targeted by TNIK-specific siRNA. The knockdown of endogenous TNIK abolished the potential of the exogenous TNIK kinase domain to activate IKKβ (Figure S3). A similar mechanism for JNK activation can be excluded because the kinase domain is dispensable for JNK signaling (see Figure 6D) and TNIK-KD overexpression does not induce JNK in HEK293 cells [1]. Taken together, these findings are in line with the concept of a role for TNIK autophosphorylation in NF-κB signaling by LMP1.

To further validate the importance of the TNIK kinase domain for canonical NF-κB signaling, we tested if overexpression of the kinase-negative mutant TNIK-KM had a dominant-negative effect on LMP1-induced NF-κB signaling in reporter assays (Figure 6F). In fact, TNIK-KM expression reduced NF-κB activation by wildtype LMP1 to almost 50%, a factor that was comparable to the effect of TNIK knockdown on LMP1-induced NF-κB (see Figure 4C). Moreover, NF-κB signaling of LMP1(AAA), which only harbors functional CTAR2, was affected by TNIK-KM but not that of LMP1Δ371–386, which solely signals via CTAR1 (Figure 6F). Thus, TNIK-KM exerted its dominant-negative effect on CTAR2-induced NF-κB signaling, confirming that the kinase activity of TNIK is critical for activation of canonical NF-κB by LMP1-CTAR2.

### TRAF6 Directly Binds TNIK and Mediates TNIK Interaction with LMP1

**Figure 7:**
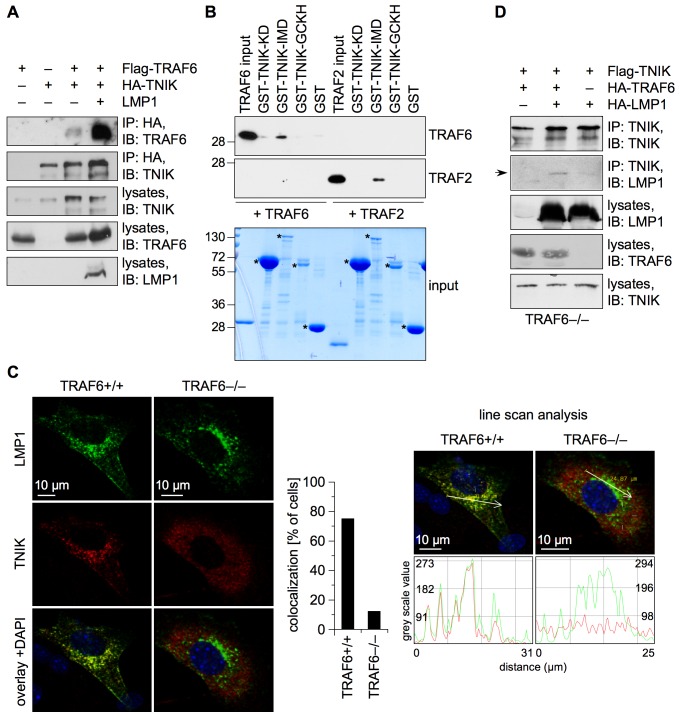
TRAF6 directly binds to TNIK and mediates TNIK recruitment to LMP1. (A) LMP1 induces interaction of TNIK and TRAF6. HEK293 cells were transfected in 10 cm cell culture dishes with 0.5 µg each of Flag-TRAF6 and HA-TNIK expression vectors, both in the presence or absence of 4 µg pSV-LMP1, as indicated. Cells were lysed and HA-TNIK was immunoprecipitated using the anti-HA (12CA5) antibody. As indicated, the following antibodies were used for immunoblot analysis of immunoprecipitations and lysates: anti-TRAF6 (H-274), anti-TNIK, and anti-LMP1 (1G6-3). IB, immunoblot; IP, immunoprecipitation. *n* = 3. (B) TRAF6 and TRAF2 bind directly to the intermediate domain of TNIK. Protein binding assays with purified proteins. Interaction of His-tagged TRAF2(311–501) and TRAF6(310–522) with GST-tagged kinase-negative kinase domain, intermediate domain, and GCKH domain of TNIK was analyzed on immunoblots with the anti-TRAF2 (C-20) and anti-TRAF6 (C-20) antibodies (upper panels). Plain GST was incorporated as a control. For immunoblots only 25 ng of the TRAF input was loaded to avoid overexposure. Input of TRAF and GST-TNIK proteins is shown in a coomassie-stained gel (lower panel). Asterisks indicate GST and GST-TNIK proteins. Apparent molecular masses are given in kDa. *n* = 2. (C) TRAF6 mediates the interaction between TNIK and LMP1. Wildtype and TRAF6−/− MEF cells were electroporated with Flag-TNIK and LMP1 vectors. Cells were immunostained for TNIK (red) and LMP1 (green) as described in Materials and Methods. Nuclei were counterstained with DAPI (blue). The graph gives the percentage of cells with co-localization of TNIK and LMP1. A line scan analysis (right panel) shows intensities of TNIK (red) and LMP1 (green) signals along the indicated arrows. LMP1 and TNIK localization peaks overlap only in wildtype but not in TRAF6−/− cells. *n* = 3. (D) Exogenous TRAF6 rescues the interaction between TNIK and LMP1 in TRAF6−/− cells. TRAF6−/− MEFs were electroporated with 5 µg each of Flag-TNIK, HA-TRAF6, and HA-LMP1 vectors as indicated. Total transfected DNA was adjusted to 20 µg with empty vector. TNIK was immunoprecipitated from cell lysates using the anti-TNIK antibody. Complex formation of LMP1 and TNIK was analyzed by immunoblotting of the IPs using the anti-TNIK and anti-LMP1 (1G6-3) antibodies. Lysates were probed with anti-LMP1 (1G6-3), anti-TRAF6 (H-274), and anti-TNIK. The arrow indicates the position of LMP1. *n* = 2.

To better understand TNIK's molecular functions in JNK and NF-κB activation and its role as bifurcation point of the two pathways, it was necessary to identify TNIK interaction partners in LMP1 signaling. The first step was to investigate how TNIK interacts with LMP1 and to characterize potential mediators of this interaction. TNIK has been shown to bind TRAF2 via its intermediate domain [1]. This finding suggested that TRAF molecules might physically couple TNIK to upstream inducers/receptors. CTAR2 signaling to JNK and IKKβ/NF-κB essentially requires TRAF6 but not TRAF2 [18],[33],[34],[38],[48]. Despite the fact that an interaction of TRAF6 with TNIK has not been described so far, we tested if TRAF6 binds to TNIK in LMP1 signaling by immunoprecipitation experiments in HEK293 cells (Figure 7A). In the absence of LMP1 a weak co-precipitation of HA-TNIK and Flag-TRAF6 was detected. Strikingly, LMP1 induced a very strong interaction of both proteins, demonstrating (i) that TRAF6 is a novel binding partner of TNIK and (ii) that interaction of both proteins is greatly enhanced upon activation (Figure 7A). The effects of LMP1 on TNIK-TRAF interaction were, however, not restricted to TRAF6. CTAR2, but not CTAR1, induced a weak but detectable interaction of TNIK with TRAF2 (Figure S4). Because studies in TRAF2-deficient cells have clearly excluded a critical function for TRAF2 in CTAR2 signaling [18],[33],[48], we concentrated our further studies on the newly identified and CTAR2-critical TNIK interaction partner TRAF6.

The TNIK intermediate domain directly binds TRAF2, as has been shown by yeast-two-hybrid assays and immunoprecipitations [1]. To determine whether TRAF6 and TNIK are also direct interaction partners, in vitro binding assays using recombinant proteins purified from bacteria were performed (Figure 7B). Indeed, the C-terminal TRAF domain of TRAF6 (amino acids 310–522) specifically bound to the immobilized GST-tagged TNIK intermediate domain. Purified TRAF2 (amino acids 311–501) was included into the experiment as a control, which also interacted with the intermediate domain of TNIK. No interaction of the two TRAFs with the TNIK kinase domain, the GCKH domain, or the GST control beads was observed. Thus, the C-terminal TRAF domain of TRAF6 directly binds to the TNIK intermediate domain.

In order to investigate whether TRAF6 acts as mediator of the interaction between TNIK and LMP1 we analyzed the subcellular localization of transiently expressed HA-TNIK and LMP1 in TRAF6-deficient and wildtype mouse embryonic fibroblasts. Confocal immunofluorescence microscopy revealed a high degree of co-localization of TNIK and LMP1 in the TRAF6+/+ cells (Figure 7C). LMP1 did not induce translocation of TNIK into the nucleus as it has been shown for Wnt signaling in intestinal cells [5]. There was no significant co-localization of LMP1 and TNIK in TRAF6−/− cells. This finding was substantiated by a grey scale line scan analysis of the microscopic images confirming that the distribution of TNIK and LMP1 displays a high degree of co-localization in wildtype cells. In contrast, the absence of TRAF6 caused a more dispersed localization of TNIK and prevented its recruitment to LMP1 (Figure 7C). This result showed that TNIK and LMP1 interact in an indirect manner and that TRAF6 is crucial for this interaction. To verify this finding by a biochemical approach we performed a rescue experiment in TRAF6−/− cells. LMP1 and Flag-TNIK were expressed in TRAF6−/− cells in the absence or presence of exogenously expressed TRAF6 (Figure 7D). LMP1 co-precipitated with Flag-TNIK only when TRAF6 was transfected. Exogenous TRAF6 expression was thus able to rescue the interaction between TNIK and LMP1 in TRAF6-deficient cells. Taken together we revealed TRAF6 as a novel direct interaction partner of the TNIK intermediate domain and as critical mediator of the interaction between TNIK and LMP1.

### TNIK Forms a Dynamic Signaling Complex with TAK1, TAB2, and IKKβ

**Figure 8:**
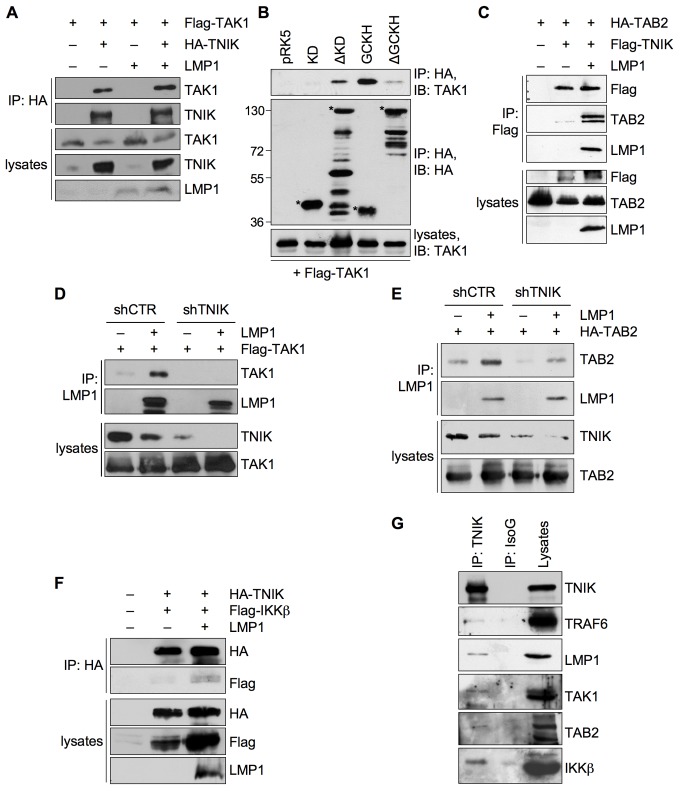
TNIK forms a dynamic signaling complex with TAK1, TAB2, and IKKβ upon LMP1 activation. (A) TAK1 interacts with TNIK independent of LMP1. HEK293 cells were transfected in 15 cm culture dishes with 5 µg of HA-TNIK and 2 µg of Flag-TAK1 vectors, both in the presence and absence of 5 µg pSV-LMP1, as indicated. HA-TNIK was immunoprecipitated with the anti-HA (12CA5) antibody. Immunoprecipitations were analyzed using anti-TNIK and anti-TAK1 antibodies. Cell lysates were stained with anti-TNIK, anti-TAK1, and anti-LMP1 (CS1-4) antibodies. *n* = 3. (B) TAK1 binds to the GCKH and intermediate domains of TNIK. HEK293 cells were transfected in 10 cm cell culture dishes with 2 µg of the indicated HA-TNIK constructs and 1 µg of Flag-TAK1 vector. HA-TNIK constructs were immunoprecipitated with the anti-HA (12CA5) antibody. Immunoprecipitations and lysates were analyzed with anti-HA (3F10) and anti-TAK1 antibodies, as indicated. Asterisks indicate TNIK proteins. Apparent molecular masses are given in kDa. *n* = 4. (C) LMP1 induces the interaction between TNIK and TAB2. HEK293 cells were transiently transfected in 15 cm culture dishes with 5 µg of HA-TAB2 and Flag-TNIK vectors in the presence or absence of 5 µg pSV-LMP1 as indicated. Flag-TNIK was immunoprecipitated using the anti-Flag (6F7) antibody. The following antibodies were used for immunoblotting: anti-Flag (6F7), anti-TAB2, anti-LMP1 (1G6-3). *n* = 3. (D) TNIK is essential for TAK1 interaction with LMP1. HEK293 cells were transfected in 15 cm culture dishes with 5 µg Flag-TAK1, 3 µg LMP1, and 7 µg each of shTNIK or shCTR vectors, as indicated. Cells were lysed 48 h post-transfection, LMP1 was immunoprecipitated using the anti-LMP1 (1G6-3) antibody, and co-precipitation of Flag-TAK1 was analyzed by immunoblotting for TAK1. TNIK knockdown and Flag-TAK1 expression was verified in total cell lysates. *n* = 3. (E) TNIK mediates the interaction between TAB2 and LMP1. HEK293 cells were transfected with the indicated vectors as described in (D). LMP1 was precipitated from cell lysates using the anti-LMP1(1G6-3) antibody. Immunoprecipitations and lysates were analyzed with the indicated antibodies. *n* = 2. (F) LMP1 induces the recruitment of IKKβ to the TNIK complex. HEK293 cells were transfected in 10 cm culture dishes with 2 µg HA-TNIK, 2 µg Flag-IKKβ, and 3 µg pSV-LMP1 as indicated. HA-TNIK was immunoprecipitated using the anti-HA (12CA5) antibody and immunocomplexes were analyzed by immunoblotting using anti-HA (12CA5) and anti-Flag (M2) antibodies. Expression of HA-TNIK, Flag-IKKβ, and LMP1 was detected in cell lysates with the anti-HA (12CA5), anti-Flag (M2), and anti-LMP1 (1G6-3) antibodies. *n* = 3. (G) TNIK signaling complex containing LMP1, TRAF6, TAK1, TAB2, and IKKβ in EBV-transformed lymphoblastoid cells. Endogenous TNIK was immunoprecipitated from LCL 721 cells with the anti-TNIK antibody. An unrelated mouse isotype IgG was used for control precipitation (IsoG). As indicated, immunoprecipitations and lysates were analyzed by immunoblotting using the anti-TNIK, anti-TRAF6 (H-274), anti-LMP1 (1G6-3), anti-TAK1, anti-TAB2, and anti-IKKβ antibodies. *n* = 2.

TAK1 interacts via TAB2 with TRAF6 to activate JNK and IKKβ/NF-κB signaling (see Introduction). Previous studies have shown that TAK1 mediates JNK signaling by LMP1, whereas the role of TAK1 in NF-κB activation is controversial [37],[40],[42]. Having defined a role for TNIK as an interaction partner of TRAF6 in JNK and canonical NF-κB signaling by LMP1, we asked whether TAK1 and TAB2 interact with TNIK as well. Indeed, TNIK and TAK1 readily interacted in HEK293 cells (Figure 8A). As the presence or absence of LMP1 had no striking effect on the affinity of both proteins we concluded that TNIK and TAK1 bind to each other constitutively. We next analyzed this interaction with regard to the TNIK domains that mediate TAK1 binding by using TNIK deletion constructs for immunoprecipitations (Figure 8B). Whereas the GCKH domain alone bound to TAK1, no interaction was detectable with the TNIK kinase domain. Deletion of the GCKH domain (HA-TNIK-ΔGCKH construct) strongly diminished the interaction with TAK1. The main TAK1 interaction site of TNIK is thus the GCKH domain and the intermediate domain contributes some binding activity as well, possibly by an indirect mechanism. It is important to note at this point that the GCKH domain of the MAP4K TNIK induces JNK signaling (see Figure 6D) and at the same time binds the critical MAP3K for this pathway, TAK1.

Co-immunoprecipitation experiments in HEK293 cells showed that TAB2 also specifically co-precipitates with TNIK (Figure 8C). However, this interaction is activation-dependent, as TAB2 did only very weakly bind to TNIK unless LMP1 was present. LMP1 co-expression induced a strong interaction of TNIK with TAB2.

Notably, TNIK is required for the interaction of TAK1/TAB2 with the LMP1 complex. The knockdown of endogenous TNIK by expression of shRNA abolished binding of TAK1 and TAB2 to LMP1 in co-immunoprecpitation experiments (Figure 8D and 8E, respectively). Thus, TNIK has an important role in the assembly of the LMP1 signalosome by acting as an interaction mediator of critical components of the complex.

We have shown that LMP1 activates IKKβ via TNIK. Therefore, we asked whether IKKβ is also a component of the TNIK signaling complex. Indeed, IKKβ also bound to TNIK, albeit only in the presence of LMP1 (Figure 8F). The interaction of TNIK with IKKβ appeared to be weaker as compared to TRAF6, TAK1, or TAB2, potentially indicating an indirect recruitment of IKKβ to TNIK. In summary, we found that TNIK forms a dynamic complex incorporating critical components of TRAF6-dependent JNK and NF-κB signaling, namely TRAF6, TAK1/TAB2, and IKKβ. TAK1 seems to be constitutively associated with TNIK, whereas the other components enter the complex after activation.

### A Complex of TNIK with LMP1, TRAF6, TAK1/TAB2, and IKKβ Exists in EBV-Transformed Human B-Cells

We sought to verify the existence of an endogenous TNIK signaling complex in lymphoblastoid cells that endogenously express LMP1. TNIK was immunoprecipitated from LCL 721 cell lysates and components of the signaling complex were analyzed by immunoblotting (Figure 8G). We found that LMP1, TRAF6, TAK1, TAB2, and IKKβ specifically bind to TNIK in LCLs, thus proving the existence of the LMP1-induced TNIK signaling complex in its native context. Taken together, our results show that the TNIK complex, which is composed of TRAF6 and LMP1 as upstream components and of TAK1/TAB2 and IKKβ as downstream mediators, is essential for JNK and canonical NF-κB activation by LMP1 in EBV-transformed human B-cells.

### TNIK Is Critical for JNK and Canonical NF-κB Signaling by CD40

Having characterized TNIK as a mediator of signal transduction by the viral pseudoreceptor LMP1, we tested a general requirement for TNIK in JNK and canonical NF-κB activation by a cellular receptor in B-cells. Because LMP1 is a functional mimic of CD40 and TRAF6 plays a pivotal role as an adapter protein for both LMP1 and CD40, we tested whether CD40 engages TNIK for signal transduction.

**Figure 9:**
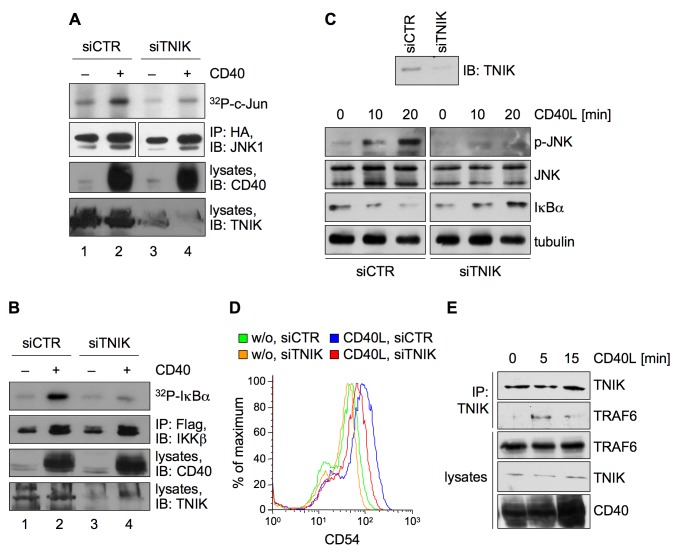
TNIK is required for JNK and canonical NF-κB signaling by CD40. (A) The knockdown of TNIK impairs CD40-induced JNK signaling in HEK293 cells. HEK293 cells were transfected in 6-well plates with TNIK or non-targeting siRNA. Subsequently, the cells were transfected with 2 µg of the CD40 expression vector pESBOS-CD40 where indicated. 1 µg of pRK5-HA-JNK1 was co-transfected. HA-JNK activity was monitored in immunocomplex kinase assays. Immunoprecipitated HA-JNK1, expression of CD40, and downregulation of TNIK was detected using the anti-JNK1, anti-CD40, and anti-TNIK antibodies. Quantification of four independent experiments: lane 1, 1.0±0.0; lane 2, 7.18±3.15; lane 3, 0.8±0.59; lane 4, 3.28±1.68. (B) IKKβ activation by CD40 is blocked by TNIK downregulation. HEK293 cells were transfected as in (A) except that Flag-IKKβ was transfected instead of HA-JNK1. Flag-IKKβ immunocomplex kinase assays were performed. Flag-IKKβ, CD40, and TNIK were detected using the indicated antibodies. Quantification of three independent experiments: lane 1, 1.0±0.0; lane 2, 8.17±1.81; lane 3, 0.6±0.16; lane 4, 3.2±1.36. (C) The knockdown of TNIK in B-cells inhibits JNK and canonical NF-κB signaling after stimulation of the endogenous CD40 receptor. BL41 cells were treated with 5 µM Accell TNIK or non-targeting siRNA for 72 h. Cells were stimulated with 500 ng/ml recombinant human CD40 ligand for the indicated times. Cell lysates were subjected to immunoblot analysis using anti-phospho-JNK, anti-JNK1, anti-IκBα, and anti-TNIK antibodies. Equal loading was verified by staining with anti-tubulin antibodies. *n* = 3. (D) TNIK mediates CD54 upregulation by CD40. BL41 B-cells were incubated with TNIK or non-targeting Accell siRNA and stimulated with 1 µg/ml CD40 ligand as indicated and described in Materials and Methods. CD54 surface levels were analyzed by flow cytometry. *n* = 2. (E) CD40 stimulation induces the interaction of TNIK and TRAF6 in B-cells. BL41 cells were stimulated with 500 ng/ml CD40 ligand. At the indicated times, cells were lysed and endogenous TNIK was immunoprecipitated by the anti-TNIK antibody. TRAF6 co-precipitation was detected by the anti-TRAF6 (H-274) antibody. Lysate controls were stained with the indicated antibodies. *n* = 3.

First we analyzed the effect of TNIK knockdown on JNK1 and IKKβ activation by CD40 in HEK293 cells. Overexpression of CD40 was sufficient to activate CD40 signaling in HEK293 cells without the need to further stimulate with CD40L (CD40 ligand). TNIK was downregulated by siRNA and cells were co-transfected with either HA-JNK or Flag-IKKβ and CD40 expression vectors. HA-JNK and Flag-IKKβ kinase assays proved that the downregulation of TNIK in fact blocked CD40-induced JNK and IKKβ activation (Figure 9A and 9B, respectively).

In order to confirm these results in human B-cells, BL41 cells were depleted of endogenous TNIK by siRNA and stimulated with recombinant soluble CD40L (Figure 9C). The knockdown of TNIK resulted in a nearly complete inhibition of CD40-induced JNK phosphorylation, demonstrating an important role of TNIK in JNK activation by CD40 also in B-cells. IκBα degradation after 10 to 20 min of CD40 stimulation indicated activation of the NF-κB pathway when cells were treated with non-targeting control siRNA. In contrast, after TNIK downregulation by siRNA the NF-κB pathway did not respond to CD40 stimulation as IκBα levels did not decrease over time (Figure 9C). These data demonstrated that TNIK is a novel and critical intermediate of endogenous CD40 signaling in human B-cells on the JNK and NF-κB axes.

CD40 stimulation activates BL41 cells, detectable as upregulation of activation markers at the cell surface such as CD54, an adhesion molecule also known as ICAM-1 and hallmark of B-cell activation [49]. CD54 upregulation by CD40 is dependent on canonical NF-κB in BL cells [50]. We tested if the knockdown of TNIK affected CD54 surface upregulation by CD40 ligand stimulation of BL41 cells. TNIK dowregulation resulted in a marked decrease of CD40-induced CD54 surface levels, demonstrating an important role for TNIK also in B-cell activation (Figure 9D).

TRAF6 is an essential signaling mediator of both LMP1 and CD40, and we have demonstrated recruitment of TRAF6 to TNIK in the context of LMP1 signaling. Therefore we asked whether CD40 stimulation can also induce an interaction between TNIK and TRAF6 in B-cells. BL41 cells were stimulated with CD40L for 0, 5, and 15 min and TNIK was immunoprecipitated and tested for TRAF6 co-precipitation. We observed that CD40 induced an interaction between endogenous TNIK and endogenous TRAF6 already 5 min after stimulation (Figure 9E). Ten minutes later the majority of TRAF6 had already dissociated from TNIK. The prompt interaction between TNIK and TRAF6 in response to CD40 stimulation demonstrates a role for the TNIK–TRAF6 complex in the context of CD40 signaling, suggesting that interaction of both molecules is a key step in signaling by LMP1 and CD40. Taken together we have identified TNIK as an important mediator of JNK and also canonical NF-κB in physiological CD40 stimulation.

## Discussion

**Figure 10:**
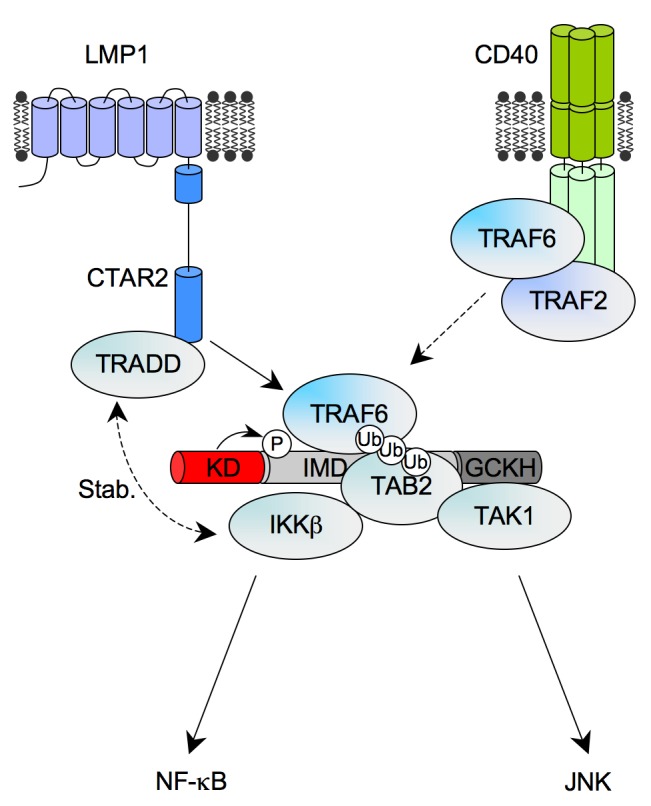
Model of TNIK's role in JNK and canonical NF-κB signaling. LMP1 induces the formation of the TNIK complex at its JNK1 and NF-κB-inducing CTAR2 domain. Interaction of TNIK with LMP1 is mediated by TRAF6, which forms an induced complex with TNIK. Also TAB2 and IKKβ are recruited upon activation, whereas TAK1 is permanently complexed with TNIK. In LMP1 signaling, IKKβ recruitment is further enhanced or stabilized by a TRADD-dependent mechanism. TNIK organizes bifurcation of the JNK and NF-κB pathways downstream of TRAF6. Auto-phosphorylation of TNIK likely constitutes an important step in transmitting signals on the NF-κB axis. JNK signaling is triggered at the GCKH domain of TNIK through TAK1/TAB2. Activation of TAK1/TAB2 further involves K63 ubiquitinylation of TRAF6, which has been demonstrated earlier. Also CD40 signals through the TRAF6/TNIK complex to JNK and IKKβ/NF-κB. For more details, see the text.

In this study we have identified and characterized the germinal center kinase family member TNIK as a novel component of the TRAF6/TAK1/TAB2/IKKβ complex. TNIK is required for JNK and canonical NF-κB signaling by the EBV oncoprotein LMP1 and its cellular counterpart CD40. According to this critical function in signaling, TNIK has an important role in mediating proliferation and survival of EBV-transformed B-cells and in physiological B-cell activation by CD40. In an unbiased functional proteomics screen TNIK was isolated as an interaction partner of the LMP1 complex in EBV-infected primary human B-cells. TNIK binding to the CTAR2 domain of LMP1 is mediated by TRAF6, a newly described direct interaction partner of TNIK. We thus report the first molecular function for TNIK's interaction with TRAF molecules. The existence of a CTAR2-induced signaling complex was revealed involving activation-dependent binding of TRAF6, TAB2, and IKKβ to TNIK. Importantly, CD40 stimulation also induces association of TNIK with TRAF6. Because TNIK's activities in JNK1 and NF-κB signaling map to different TNIK domains, we propose a model in which TNIK orchestrates bifurcation and signal transmission of both pathways at the level of the TRAF6/TAK1/TAB2/IKKβ complex (Figure 10). Our discovery that TNIK is a new key player in TRAF6-dependent JNK and canonical NF-κB signaling significantly extends the current concept of molecular regulation of these pathways.

LMP1 is constitutively active and closely mimics the TNFR family member CD40 in B-cell activation [11]. Despite differences in the molecular composition and efficiency of their signaling complexes, LMP1 and CD40 share similarities with regard to the engagement of TRAF molecules and the pattern of activated signal transduction pathways [14]. TRAF6 plays a pivotal role in canonical NF-κB and JNK signaling by both receptors [33],[37],[38],[51]. Both LMP1 and CD40 induce association of TNIK with TRAF6, whose interaction is direct and involves the TRAF domain of TRAF6 and the intermediate domain of TNIK. This interaction couples TNIK to the upstream receptor. Apart from TRAF6, TNIK can also interact with TRAF2. However, TRAF2 is dispensable for JNK activation, IκB-dependent NF-κB signaling, and p65 nuclear translocation by LMP1 and can thus be excluded as an essential mediator of TNIK interaction with CTAR2 [33],[34],[48]. The CTAR2-induced association of TNIK and TRAF2 detected here might have a non-essential accessory role in CTAR2 signaling. Also the CTAR2-interacting factor TRADD is unlikely to play a central role in TNIK recruitment because it is exclusively involved in IKKβ/NF-κB activation by CTAR2 but not in JNK signaling, whereas TNIK is required for both signaling pathways [35]. Upon activation by LMP1, TAB2 and IKKβ are recruited to TNIK. This observation of activation-induced complex formation is in line with findings that the dynamic association of TAB2 with TRAF6 and TAK1 also occurs in other pathways, for instance in interleukin-1 signaling [52]. TAK1, in contrast, interacts constitutively with TNIK. We therefore propose that upon activation the TNIK-TAK1 complex is recruited to the CTAR2 domain via TRAF6 and recruits additional downstream signaling mediators such as TAB2 and IKKβ. The signaling complex is likely further stabilized by TRADD, which is involved in the recruitment of IKKβ to the LMP1 complex [35]. The TRADD-dependent stabilization of the complex at CTAR2 might in part explain the more efficient signaling complex of LMP1 as compared to CD40. In contrast to LMP1, CD40 induction of JNK and IκB phosphorylation involves TRAF2 in B-cells [53],[54]. Moreover, TRAF2 is involved in TRAF6 recruitment to the distal TRAF binding site of CD40 that induces JNK and canonical NF-κB signaling [45]. For these reasons, a more pronounced role of TRAF2 in TNIK interaction with CD40 seems feasible. Future studies will have to dissect the precise role of TRAF family members in coupling TNIK to CD40. However, because CD40 induces a rapid interaction of TNIK with TRAF6, we suggest a critical role of TRAF6 in this process, which could involve additional members of the TRAF family.

The TNIK-TRAF6-TAK1/TAB2-IKKβ complex mediates activation of the canonical NF-κB and JNK pathways. TNIK is, to our knowledge, the only known protein within this complex whose activities on the NF-κB and JNK axes are clearly allocated to separate domains of the same protein. NF-κB activation depends on the kinase and intermediate domains of TNIK, whereas signaling to JNK only involves the GCKH domain. Thus, TNIK seems to constitute the molecular organizer of JNK and NF-κB bifurcation. It has been shown that purified TAK1 together with TAB1/TAB2 is sufficient to phosphorylate and thus activate IKKβ in a test tube. This reaction further depends on TRAF6 and Ubc13/Uev1A [46],[55]. Our results demonstrate that TNIK is additionally required to assemble, organize, and activate the holocomplex in vivo and to recruit the complex to the receptor (here: LMP1) by acting as an adapter and scaffolding protein. Due to its interaction with TRAF molecules TNIK is likely involved in the specific coupling of the TAK1-TAB2 and IKK modules to distinct receptors.

The GCKH domain of the MAP4kinase TNIK mediates JNK activation and is also the main interaction site of the JNK-inducing MAP3kinase TAK1, suggesting that TNIK acts directly upstream of TAK1 in the signaling cascade. However, TNIK's kinase activity is dispensable for JNK activation. Similar to other germinal center kinases, TNIK may facilitate MAP3kinase activation by inducing conformational changes that induce MAP3K autophosphorylation and thus activation of the MAP3K [2],[56].

In contrast to the JNK pathway, activation of IKKβ by TNIK critically depends on the kinase activity of TNIK. IKKβ itself does not appear to be a TNIK substrate because we could not detect direct IKKβ phosphorylation by TNIK (unpublished data). However, LMP1 induces TNIK's activity to phosphorylate itself, supporting a role for TNIK autophosphorylation in signaling. The fact that exogenous TNIK-KD is incapable of activating IKKβ in the absence of endogenous TNIK suggests that phosphorylation is important for NF-κB activation but localizes to a domain other than the kinase domain of TNIK. A similar mechanism for JNK signaling is not likely because TNIK-KD is dispensable for JNK activation and TNIK-KD overexpression does not induce JNK (see Figure 6D) [1]. Multiple phosphorylation sites cluster within TNIK's intermediate domain (www.phosphosite.org) [1],[3], which is the likely target of TNIK's autophosphorylation. Phosphorylation of TNIK seems to have different effects. One study reported that autophosphorylated TNIK is found in the cytoskeletal fraction, where it mediates disassembly of F-actin [3]. Wnt/β-catenin signaling leads to the phosphorylation of TNIK at serine 764 and translocation of TNIK into the nucleus, where it interacts with TCF4 to mediate activation of Wnt target genes [5],[6]. Using a phophosite-specific antibody we observed that LMP1 does not induce phosphorylation of TNIK at serine 764 (unpublished data), which is consistent with the fact that LMP1 does not induce Wnt signaling [57]. We consider the relevance of a different phosphorylation site within TNIK in the context of canonical NF-κB signaling. It will be the focus of future studies to identify TNIK autophosphorylation sites as well as possible other TNIK substrates in the NF-κB pathway, for instance by phosphoproteomics.

Due to its universal expression pattern we envision that TNIK functions as a mediator of TRAF-dependent JNK and NF-κB activation in various tissues and cell types. So far, TNIK has been described to regulate neurite growth and neuronal morphology in the brain and to be involved in the activation of Wnt target genes in intestinal crypt cells [5],[6],[8],[58]. Here we extend the functions of TNIK to lymphocytes. Our data indicate important roles for this kinase in B-cell function, immunity, and cancer. JNK and NF-κB have pivotal roles in physiological activation and oncogenic transformation of B-cells [45],[59],[60]. LMP1 and CD40 are involved in various malignant diseases of the hematopoietic system, such as Hodgkin's and non-Hodgkin's lymphoma, post-transplant lymphoproliferative disease, or chronic lymphocytic leukemia, as well as in non-hematopoietic cancers such as nasopharyngeal carcinoma or renal carcinoma [60]–[62]. Notably, the LMP1-induced canonical NF-κB and JNK pathways are known to be essential for LMP1-mediated B-cell transformation by activation of anti-apoptotic and cell cycle-promoting signals [31],[32],[63]. Accordingly, we showed that TNIK is essential for lymphoblastoid proliferation and survival. CD40-induced NF-κB, which also involves TNIK, protects cells from apoptosis in some low-grade B-cell malignancies and promotes cell transformation of epithelial cells, for instance in breast cancer [62],[64]. Thus, our data implicate TNIK in LMP1- and CD40-induced cancer and indicate the potential of TNIK as a future target for therapy of EBV and CD40-associated malignancies.

## Materials and Methods

### Plasmids and Cloning

pSV-LMP1, pSV-LMP1Δ194–386 lacking the LMP1 signaling domain, pCMV-HA-LMP1, pCMV-HA-LMP1Δ194–386, pCMV-HA-LMP1(AAA) harboring a P(204)xQxT to AxAxA mutation within CTAR1, pCMV-HA-LMP1Δ371–386 lacking the 16 C-terminal amino acids of CTAR2, the double mutants pCMV-HA-LMP1(AAA, Δ371–386) and pCMV-HA-LMP1(AAA, Y384G), as well as the fusion constructs pCMV-HA-LMP1-TNFR1ΔDD and pCMV-HA-LMP1-TNFR1-CTAR2 (alternative name: pCMV-HA-LMP1-TNFR1-LTB) have been described [34],[35],[38]. pESBOS-CD40, pCMV-HA-TAB2, pRK-TRAF2, pRK5-HA-JNK1, and pcDNA3-Flag-IKKβ have been described [30],[35],[40]. pCMV-HA-LMP1-liTEV-CT was cloned by a PCR approach on the basis of pCMV-HA-LMP1. A flexible linker sequence (AGASGGAGASGG) and a TEV cleavage site (ENLYFQG) were inserted between amino acids Y186 and H187 of LMP1. To generate pRK5-HA-TNIK, pRK5-HA-TNIK-KD, pRK5-HA-TNIK-GCKH, pRK5-HA-TNIKΔKD, and pRK5-HA-TNIKΔGCKH, TNIK sequences were amplified from human TNIK cDNA [1] and HA-tagged by PCR, and subsequently cloned into pRK5. The vector pRK5-HA-TNIK(KM) harboring a K54R mutation within the TNIK kinase domain was subcloned from pYCI-TNIK(KM) [1]. pRK5-Flag-TNIK was generated by PCR on the basis of pRK5-HA-TNIK. pRK5-HA-TNIK-KDwob was cloned by a PCR approach on the basis of pRK5-HA-TNIK-KD. pRK5-HA-TNIK-KDwob harbors silent wobble mutations at the nucleotide level to eliminate the targeting sequence of human Dharmacon TNIK ON TARGETplus SMARTpool siRNA J-004542-10 (targeting sequence: GAACATACGGGCAAGTTTA). pRK5-HA-TRAF6 and pRK5-Flag-TRAF6 were generated by PCR approaches based upon human TRAF6 cDNA [38]. pRK5-Flag-TAK1 was cloned by a PCR approach using a TAK1 cDNA [40]. Bacterial expression vectors for glutathione-S-transferase (GST)-fused TNIK domains were generated by subcloning TNIK-KD(KM), TNIK-IMD, and TNIK-GCKH sequences from pRK5 background into pGEX2T (GE Healthcare). The C-terminal TRAF domains of human TRAF2 (amino acids 311–501) and TRAF6 (amino acids 310–522) were cloned by PCR approaches from cDNAs [34],[38] into the pET17b vector (Novagen) with an N-terminal His-tag. All constructs were verified by sequencing. Detailed cloning strategies and PCR primer sequences can be made available upon request.

### Cell Culture Methods

The EBV-positive lymphoblastoid B-cell lines LCL 721, EREB2-5, and LCL3 have been described [35],[65],[66]. The generation of LCL-TEV.5 cells is described herein. HEK293 human embryonic kidney cells, the human EBV-negative Burkitt's lymphoma B-cell line BL41 [67], and all lymphoblastoid cell lines were grown in RPMI full medium (Invitrogen) supplemented with 10% fetal calf serum (Biochrom AG). EREB2-5 cells were additionally kept in the presence of 1 µM β-estradiol to activate the conditional ER-EBNA2 transcription factor that drives EBV latent genes required for proliferation of EREB2-5 cells [66]. Wildtype and TRAF6−/− mouse embryonic fibroblasts [51] were grown in DMEM (Invitrogen) supplemented with 10% fetal calf serum. BL41 cells were stimulated with the indicated amounts of human recombinant soluble CD40 ligand (Source BioScience).

### RNA Interference

HEK293 cells were seeded in 6-well plates and transfected twice within 24 h with 100 nM of human ON TARGETplus SMARTpool TNIK siRNA (pool of four siRNAs J-004542-10 to 13, Dharmacon) or corresponding ON TARGETplus non-targeting control siRNA using the Dharmafect transfection reagent according to the manufacturer's protocol. The cells were transfected 24 h later with the indicated plasmids using Polyfect transfection (Qiagen) and analyzed 24 h after the last transfection. To achieve TNIK knockdown in larger cell culture dishes, HEK293 cells were co-transfected with pSM2-shTNIK (RHS1764-949310, Open Biosystems), an expression vector for short hairpin RNA targeting TNIK, or the non-targeting control vector pSM2-shControl (RHS1707-OB, Open Biosystems) as indicated in the figure legends. LCL 721, EREB2-5, and BL41 cells were incubated with 5 µM Accell SMARTpool TNIK siRNA (pool of four Accell siRNAs J-004542-18 to 21, Dharmacon) or Accell non-targeting pool siRNA for 72–96 h in serum-free Accell delivery medium (Dharmacon). Subsequently, B-cells were lysed in Laemmli-DTT buffer (25 mM Tris-HCl pH 6.8, 1% SDS, 5% glycerine, 25 mM DTT) for immunoblotting or analyzed as indicated.

### Generation of Recombinant Maxi-EBV and Infection of Primary Human B-Cells

Recombinant maxi-EBV p2089-HA-LMP1-liTEV-CT was generated as previously described [11],[68]. In brief, HA-LMP1-liTEV-CT sequences were subcloned from pCMV-HA-LMP1-liTEV-CT into the shuttle vector p2167.1 to transfer HA-LMP1-liTEV-CT into the context of the viral LMP1 locus, HA-LMP1-liTEV-CT replacing the wildtype LMP1 gene. Homologous recombination of the shuttle vector with the p2089 wildtype maxi-EBV bacterial artificial chromosome (BAC) in *E. coli* DH10B resulted in p2089-HA-LMP1-liTEV-CT. The packaging cell line TR-2/293 was transfected with p2089-HA-LMP1-liTEV-CT DNA and virus production was induced by transfection with expression vectors for the EBV genes BALF4 and BZLF1 as described [68]. For B-cell infection, primary B-cells were prepared from human adenoids and plated together with 2089-HA-LMP1-liTEV-CT virus supernatant on a feeder layer of γ-irradiated WI38 cells in 96-well plates as described [68]. Outgrowing lymphoblastoid LCL-TEV clones were further propagated and analyzed. Expression of HA-LMP1-liTEV-CT and absence of wildtype LMP1 were confirmed by RT-PCR and immunoblotting.

### Proteomics and Mass Spectrometry

5×10^8^ lymphoblastoid cells per sample were lysed in 15 ml of IP-lysis buffer (150 mM NaCl, 50 mM HEPES pH 7.5, 5 mM EDTA, 0.1% NP-40, 0.5 mM sodium orthovanadate, 0.5 mM NaF, 0.5 mM sodium molybdate, Roche complete proteinase inhibitor) and cleared by centrifugation at 16,000 g. To immunoprecipitate HA-LMP1-liTEV-CT the lysates were incubated with the anti-HA (12CA5) antibody (mouse, Roche) covalently coupled to protein-A-sepharose beads (Roche) by treatment with 20 mM dimethyl pimelimidate (Fluka). Immunoprecipitations were washed three times with IP-lysis buffer, and TEV protease cleavage was performed in TEV buffer (50 mM Tris-HCl pH 8.0, 0.5 mM EDTA, 1 mM dithiothreitol, 0.1% NP-40) for 4 h at 16°C. Subsequently the beads were removed by centrifugation at 500 g for 5 min to elute the released LMP1 signaling domain together with interacting proteins. Small aliquots were analyzed by immunoblotting and the remaining samples were further processed for mass spectrometry analysis. To reduce complexity of the samples, the eluate was separated on a 12.5% SDS gel and subdivided into three parts by excising coomassie-stained gel slices containing proteins in the range of <30 kDa, 30–70 kDa, or >70 kDa, respectively. After incubation of the gel slices in ABC buffer (50 mM ammonium carbonate, 30% acetonitrile), proteins were reduced by dithiothreitol and alkylated by iodoacetamide within the gel. After tryptic digestion the peptides were eluted from the gel in elution buffer (80% acetonitrile, 1% trifluoroacetic acid) and dried under vacuum. For LC-MALDI analysis of the complex mixture, peptides were dissolved in 3% acetonitrile and 0.5% trifluoroacetic acid, desalted, and subsequently separated by nano HPLC (Ultimate II HPLC, manufactured by Dionex/LC-Packings) on a C18 column using an acetonitrile gradient (5% to 80% acetonitrile, 0.08% trifluoroacetic acid) for elution. Eluted peptides were spotted with a Probot LC-MALDI spotting system (Dionex) onto a matrix-assisted laser desorption/ionization (MALDI) target with cyano-4-hydroxycinamonic acid as matrix. MALDI-TOF-TOF mass spectrometry of the peptides was performed using the Applied Biosystems (ABI) proteomics analyzer 4700. MS spectra were analyzed by the GPS 3.5 explorer software (Applied Biosystems). SwissProt databank search was performed using the Mascot algorithm.

### Immunoprecipitation, Immunoblotting, and Antibodies

HEK293 cells were seeded in cell culture dishes and transfected at 70% confluence with the indicated plasmids using the Polyfect transfection reagent according to the manufacturer's protocol (Qiagen). Twenty-four hours post-transfection, cells were lysed in IP-lysis buffer (see above). Lymphoblastoid or BL41 cells were lysed in IP-lysis buffer at a total protein concentration of 1 mg/ml. Immunoprecipitations from B-cells were performed with 3 to 5 mg of total protein per sample. Proteins were precipitated using antibodies that had been covalently coupled to protein-G-sepharose beads (GE Healthcare). The following antibodies were used for immunoprecipitation: Flag (6F7) (Sigma), HA (12CA5) (Roche), LMP1 (1G6-3) (provided by Elisabeth Kremmer) [69], and TNIK (BD Biosciences). After immunoprecipitation beads were washed four times with IP-lysis buffer and precipitated proteins were analyzed by SDS-PAGE and immunoblotting using standard protocols. The following primary antibodies were used for immunoblotting: Flag (6F7), Flag (M2) (Sigma); HA (12CA5), HA (3F10) (Roche); LMP1 (1G6-3); LMP1 (CS1-4) (Dianova); TNIK (BD Biosciences); CD40 (C-20), IKKβ (H-470), JNK1 (C-17), SAM68 (C-20), TAB2 (H-300), TAK1 (M-579), TRAF2 (C-20), TRAF3 (C-20), TRAF6 (H-274), TRAF6 (C-20), and tubulin (B-5-1-2) (Santa Cruz Biotechnology); and phospho-SAPK/JNK (Thr183/Tyr185), phospho-IκBα (Ser32) (14D4), p52/p100 (18D10), and p65 (C22B4) (New England Biolabs). Horseradish peroxidase-coupled secondary antibodies were purchased from New England Biolabs.

### Nuclear Shift Assays

HEK293 cells were lysed 24 h post-transfection for overexpression studies and 48 h post-transfection for RNAi experiments. Cells were recovered from 10 cm cell culture dishes and washed twice in PBS at 4°C. Cells were lysed in 100 µl of swelling buffer (10 mM HEPES pH 7.7, 10 mM KCl, 2 mM MgCl_2_, 0.1 mM EDTA) on ice for 10 min. Subsequently, 0.65% NP40 was added, and the samples were incubated on ice for 1 min and centrifuged for 1 min at 16,000 g. The supernatant representing the cytosolic fraction was collected, and the pellet was washed once with swelling buffer. To retrieve the nuclear fraction the pellet was lysed in 50 µl of nuclear extraction buffer (50 mM HEPES pH 7.7, 50 mM KCl, 300 mM NaCl, 0.1 mM EDTA, 10% glycerol) and insoluble debris was removed by centrifugation. The samples were further analyzed for NF-κB proteins and the marker proteins tubulin (cytoplasm) and SAM68 (nucleus) by immunoblotting.

### Immunocomplex Kinase Assays

Immunocomplex kinase assays were essentially performed as described [38]. In brief, HEK293 cells were seeded into 6-well plates and transiently cotransfected with 2 µg each of the indicated constructs and 1 µg of pRK5-HA-JNK1 for JNK1 kinase assays or pcDNA3-Flag-IKKβ for IKKβ kinase assays using Polyfect transfection. Twenty-four hours post-transfection, cells were lysed in IP-lysis buffer and HA-JNK1 or Flag-IKKβ was immunoprecipitated overnight at 4°C using immobilized anti-HA (3F10) (Roche) or anti-Flag (6F7) (Sigma) antibodies. Beads were washed twice with IP-lysis buffer and twice with kinase reaction buffer (20 mM Tris-HCl pH 7.4, 20 mM NaCl, 10 mM MgCl_2_, 1 µM DTT, 2 µM ATP). In vitro kinase assays were subsequently performed in the presence of 10 µCi γ-^32^P-ATP and 2 µg of the recombinant purified substrates GST-c-Jun or GST-IκBα, respectively, for 25 min at 26°C. Substrates were included in the reaction buffer mix and their concentrations were not rate limiting for the phosphorylation reaction at the given reaction conditions [70]. For TNIK autophosphorylation assays, HA-TNIK was immunoprecipitated using the anti-HA (12CA5) antibody, beads with precipitated HA-TNIK were washed thoroughly, and the kinase reaction was performed in kinase reaction buffer lacking any other substrate. Kinase reactions were terminated by denaturating samples in Laemmli-DTT buffer. Subsequently samples were subjected to SDS-PAGE and autoradiography. Radioactive signals were quantified using the Fuji FLA-5100 phosphoimager.

### Cell Proliferation, Apoptosis, and Flow Cytometry

For cell proliferation assays, 2×10^4^ EREB2-5 cells per well of a 96-well plate were seeded at day zero in triplicates in Accell delivery medium supplemented with 1 µM β-estradiol and 5 µM Accell SMARTpool TNIK or Accell non-targeting siRNA. Proliferation was monitored at the indicated times by 3-(4,5-dimethylthiazol-2-yl)-2,2,5-diphenyl tetrazolium bromide (MTT) conversion as described [11]. Apoptosis was assayed by staining of the cells with propidium iodide (PI) and Cy5-labeled Annexin V using the Apoptosis Detection Kit (Biocat) and subsequent flow cytometry analysis with the Becton Dickinson FACSCalibur flow cytometer. For detection of CD40-induced CD54 surface expression, 5×10^4^ BL41 cells were seeded per well of a 24-well plate in Accell delivery medium supplemented with 5 µM Accell siRNA. At day 1 and 2, the cells were stimulated with 1 µg/ml CD40 ligand or left untreated in the presence of 2% fetal calf serum. At day 3, surface CD54 was stained with an APC-conjugated anti-CD54 antibody (ImmunoTools) and detected by flow cytometry. Flow cytometry data were analyzed with the FlowJo software (TreeStar).

### Immunofluorescence Confocal Microscopy

1.5×10^6^ mouse embryonic fibroblasts were electroporated using a BioRad Gene Pulser II at 240 V and 950 µF with 2 µg each of pSV-LMP1 and pRK5-Flag-TNIK. Total transfected DNA was adjusted to 20 µg with empty vector. After transfection cells were seeded onto glass coverslips and cultivated overnight. Cells were subsequently fixed with 2% paraformaldehyde (Merck) for 15 min, permeabilized with 0.15% Trtion X-100 (Sigma) in PBS three times for 5 min, and then blocked three times for 10 min with blocking solution (PBS, 1% bovine serum albumin, 0.15% glycine). Cells were then incubated with the primary antibody in blocking solution for 2 h at room temperature. After washing once with PBS and twice with PBS containing 0.15% Triton X-100 cells were blocked for 7 min in blocking solution. Subsequently cells were incubated with secondary antibody diluted 1∶200 in blocking solution for 45 min at room temperature. The following primary antibodies were used: TNIK (mouse, BD Biosciences) and LMP1 (rat, 1G6-3). The following secondary antibodies were used: CY3-conjugated goat-anti-mouse IgG (H+L) and FITC-conjugated goat-anti-rat IgG (H+L) (both: Dianova). Images were acquired with a Leica TCS SP2 confocal laser scanning microscope fitted with a 63×1.4 HCX Plan Apo blue objective. The acquired digital images were deconvoluted and evaluated with Huygens Essential Suite 3.2 software (Scientific Volume Imaging). Colocalization events were further analyzed with grey scale signal intensity line scans.

### Reporter Assays

HEK293 cells were transfected in 6-well plates with the indicated constructs and 5 ng of the NF-κB luciferase reporter 3xκBLuc [28] together with 50 ng of a pPGK-Renilla housekeeping gene reporter construct using Polyfect transfection (Quiagen). Twenty-four hours post-transfection, cells were lysed in reporter lysis buffer and firefly and renilla luciferase activities were measured using the Dual-Luciferase reporter assay kit (Promega). Luciferase activities were normalized for renilla activities to standardize for transfection efficiency.

### Protein Purification and In Vitro Binding Assays

His-tagged TRAF domains of TRAF2 (amino acids 311–501) and TRAF6 (amino acids 310–522) were expressed in *E. coli* BL21 Codon Plus RIPL cells (Stratagene) from pET17b vectors. Protein expression was induced by induction at an OD_600_ of 0.8 with 0.1 mM isopropyl-β-D-1-thiogalactopyranoside at 20°C overnight. Bacteria were lysed by sonication in 50 mM phosphate buffer, pH 8.0, supplemented with 10 mM imidazole, 300 mM NaCl, 1 mg/ml lysozyme, and Roche complete proteinase inhibitor cocktail. Cleared lysates were incubated with Ni^2+^-NTA agarose (Qiagen) to bind His-tagged TRAF proteins. Subsequently, His-tagged proteins were washed with 50 mM phosphate buffer, pH 8.0, 300 mM NaCl and increasing imidazole concentrations (20 to 100 mM), and eluted from Ni^2+^-NTA agarose with 50 mM phosphate buffer, pH 7.4, 300 mM NaCl, and 500 mM imidazole. Eluted TRAF proteins were further purified by gel filtration on a DextraSEC PRO10 column (Applichem) and the buffer was exchanged to TBS, pH 7.4, 20% glycerol. Proteins were either directly used for experiments or stored at −20°C for up to 4 wk. For in vitro protein binding assays, 1 µg of His-TRAF2(311–501) or His-TRAF6(310–522) were incubated for 1 h at 4°C in 500 µl TBS, pH 7.4, 0.1% (w/v) BSA, 0.5% Tween 20, with immobilized GST-TNIK-KD(KM), GST-TNIK-IMD, GST-TNIK-GCKH or GST, purified from *E. coli* and coupled to glutathione sepharose (GE Healthcare). Beads were washed 3 times with TBS containing 0.1% Tween 20 and bound His-TRAF proteins were analyzed by immunoblotting.

## Supporting Information

Figure S1 **HA-LMP1-liTEV-CT induces signaling.** HEK293 cells were transfected with expression vectors for HA-LMP1 and HA-LMP1-li-TEV-CT. HA-LMP1(AAA, Y384G) harboring mutated CTAR1 and CTAR2 domains served as null control. Co-transfected HA-JNK1 was immunoprecipitated and in vitro JNK1 assays were performed using GST-c-Jun as a substrate. Immunoprecipitated HA-JNK1 was detected using the anti-JNK1(C-17) antibody, and the expressed LMP1 constructs were detected by the anti-LMP1(CS1-4) antibody.
(TIF)

Figure S2 **Effects of TNIK knockdown on EREB2-5 morphology.** Microscopic images of the experiment shown in Figure 5B taken at day 3 after TNIK knockdown. Two representative images are shown each for Accell siTNIK and Accell siCTR treatment. The siCTR samples show typical LCL clusters containing mostly healthy and rounded cells. In contrast, the knockdown of TNIK caused the appearance of many apoptotic or dead cells and the disintegration of LCL clusters. Arrows indicate examples of dead cells.
(TIF)

Figure S3 **The exogenous TNIK kinase domain requires endogenous TNIK to activate IKKβ in HEK293 cells.** HEK293 cells were transfected in 6-well plates with TNIK siRNA or non-targeting siRNA. Subsequently, 2 µg of pRK5-HA-TNIK-KDwob or empty vector were co-transfected with 1 µg Flag-IKKβ and IKKβ kinase assays were performed. pRK5-HA-TNIK-KDwob expresses the wildtype TNIK kinase domain but is not targeted by TNIK siRNA. As expected, HA-TNIK-KDwob, detected by the anti-HA (3F10) antibody, was unaffected by TNIK siRNA, whereas endogenous TNIK, stained by the anti-TNIK antibody, was downregulated.
(TIF)

Figure S4 **LMP1-CTAR2 induces an interaction between TNIK and TRAF2.** HEK293 cells were transfected in 10 cm cell culture dishes with 0.5 µg each of HA-TNIK wildtype, pRK-TRAF2, or 3 µg each of the HA-LMP1 constructs as indicated. Cells were lysed and TNIK was precipitated using the anti-TNIK antibody. Lysates and immunoprecipitations were analyzed by immunoblotting with the anti-TRAF2, anti-TNIK, and anti-LMP1 (1G6-3) antibodies.
(TIF)

Table S1 **TNIK peptides identified by mass spectrometry in the TEV eluate of HA-LMP1-liTEV-CT immunoprecipitated from LCL-TEV.5 cells.**
(DOC)

Table S2 **TNIK identification by mass spectrometry.** The significance threshold for Mascot search (MOWSE score *p* value 0.05) was 28 and corresponds to a protein score confidence interval (C.I.) of 95%.
(DOC)

## Abbreviations

AnV: annexin V
BL: Burkitt's lymphoma
CTAR: C-terminal activator region
EBV: Epstein-Barr virus
GCK: germinal center kinase
GCKH domain: germinal center kinase homology domain
GST: glutathione-S-transferase
HA: hemagglutinin
IκB: Inhibitor of κB
IKK: IκB kinase
IMD: intermediate domain
IRF7: interferon regulatory factor 7
JNK: c-Jun N-terminal kinase
KD: kinase domain
LC: liquid chromatography
LCL: lymphoblastoid cell line
LMP1: latent membrane protein 1
MALDI-TOF: matrix-assisted laser desorption/ionization-time of flight
MAP3K: MAP kinase kinase kinase
MAPK: mitogen-activated protein kinase
MEF: mouse embryonic fibroblast
MS: mass spectrometry
MTT: 3-(4,5-dimethylthiazol-2-yl)-2,2,5-diphenyl tetrazolium bromide
NF-κB: nuclear factor-κB
PAK: p21-activated kinase
PI: propidium iodide
PGK: phosphoglycerate kinase
PI3-kinase: phosphatidylinositol 3-kinase
Ste20: sterile 20
TAB: TAK1-binding protein
TAK1: transforming growth factor β-associated kinase 1
TEV: tobacco etch virus
TNFR: tumor necrosis factor-receptor
TRAF: TNFR-associated factor
TNIK: TRAF2- and Nck-interacting kinase
TRADD: TNFR-associated death domain protein
WHO: World Health Organization
