# Supplementary figures and images for: The Germinal Center Kinase TNIK Is Required for Canonical NF-κB and JNK Signaling in B-Cells by the EBV Oncoprotein LMP1 and the CD40 Receptor
Source: PLoS Biol. 2012 Aug 14;10(8):e1001376. doi: 10.1371/journal.pbio.1001376 (PMC3419181; doi:10.1371/journal.pbio.1001376)

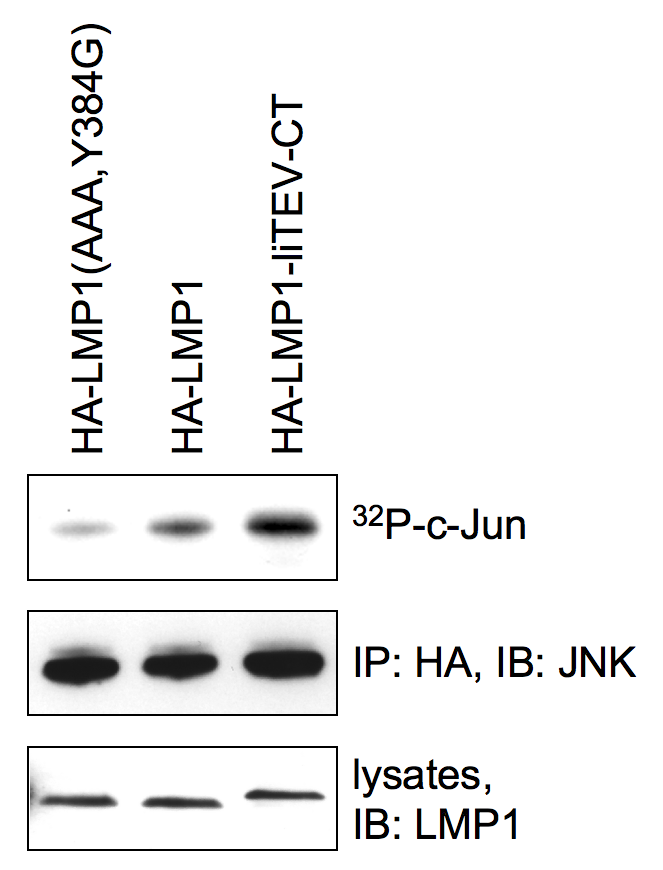

Supplement: Figure S1 — HA-LMP1-liTEV-CT induces signaling. HEK293 cells were transfected with expression vectors for HA-LMP1 and HA-LMP1-li-TEV-CT. HA-LMP1(AAA, Y384G) harboring mutated CTAR1 and CTAR2 domains served as null control. Co-transfected HA-JNK1 was immunoprecipitated and in vitro JNK1 assays were performed using GST-c-Jun as a substrate. Immunoprecipitated HA-JNK1 was detected using the anti-JNK1(C-17) antibody, and the expressed LMP1 constructs were detected by the anti-LMP1(CS1-4) antibody. (TIF) [file pbio.1001376.s001.tif]

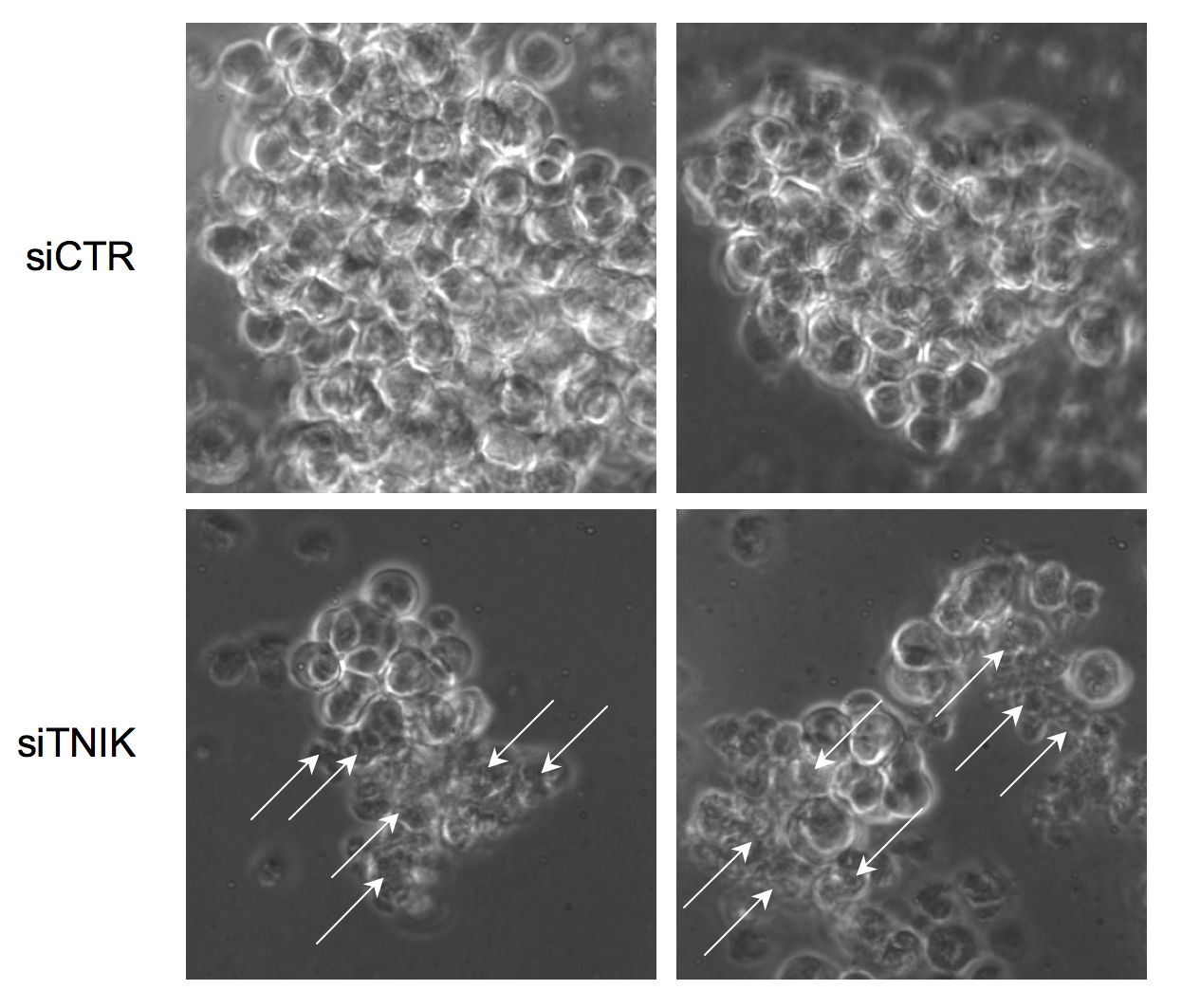

Supplement: Figure S2 — Effects of TNIK knockdown on EREB2-5 morphology. Microscopic images of the experiment shown in Figure 5B taken at day 3 after TNIK knockdown. Two representative images are shown each for Accell siTNIK and Accell siCTR treatment. The siCTR samples show typical LCL clusters containing mostly healthy and rounded cells. In contrast, the knockdown of TNIK caused the appearance of many apoptotic or dead cells and the disintegration of LCL clusters. Arrows indicate examples of dead cells. (TIF) [file pbio.1001376.s002.tif]

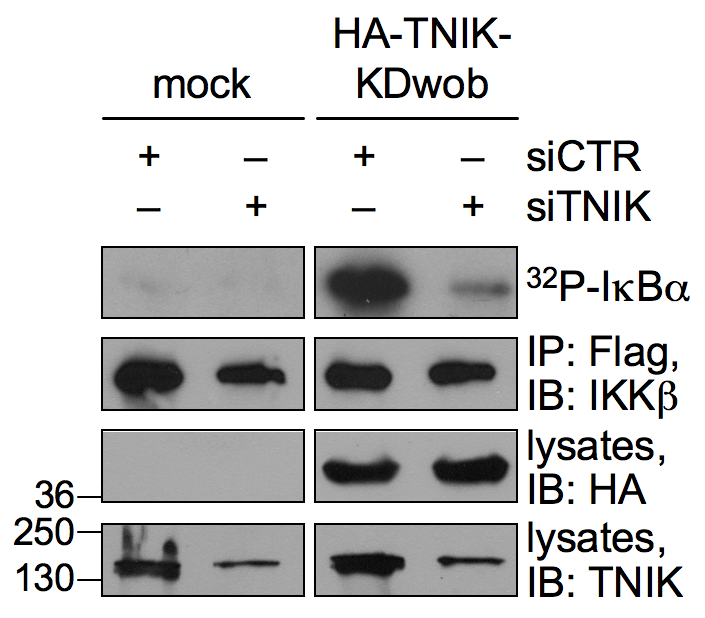

Supplement: Figure S3 — The exogenous TNIK kinase domain requires endogenous TNIK to activate IKKβ in HEK293 cells. HEK293 cells were transfected in 6-well plates with TNIK siRNA or non-targeting siRNA. Subsequently, 2 µg of pRK5-HA-TNIK-KDwob or empty vector were co-transfected with 1 µg Flag-IKKβ and IKKβ kinase assays were performed. pRK5-HA-TNIK-KDwob expresses the wildtype TNIK kinase domain but is not targeted by TNIK siRNA. As expected, HA-TNIK-KDwob, detected by the anti-HA (3F10) antibody, was unaffected by TNIK siRNA, whereas endogenous TNIK, stained by the anti-TNIK antibody, was downregulated. (TIF) [file pbio.1001376.s003.tif]

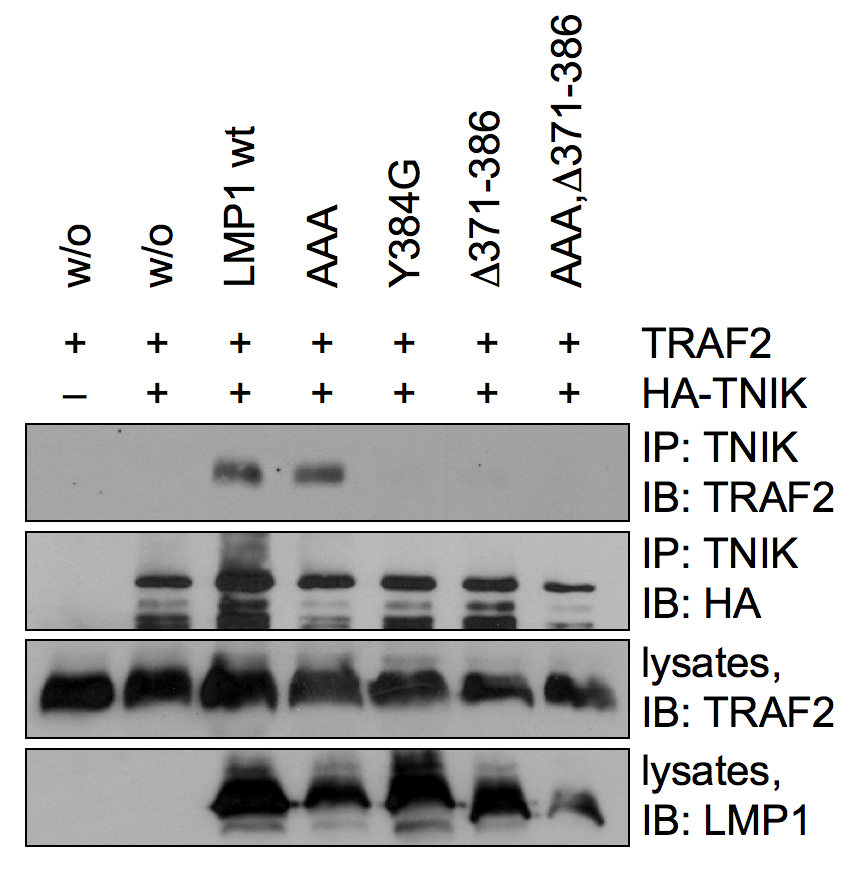

Supplement: Figure S4 — LMP1-CTAR2 induces an interaction between TNIK and TRAF2. HEK293 cells were transfected in 10 cm cell culture dishes with 0.5 µg each of HA-TNIK wildtype, pRK-TRAF2, or 3 µg each of the HA-LMP1 constructs as indicated. Cells were lysed and TNIK was precipitated using the anti-TNIK antibody. Lysates and immunoprecipitations were analyzed by immunoblotting with the anti-TRAF2, anti-TNIK, and anti-LMP1 (1G6-3) antibodies. (TIF) [file pbio.1001376.s004.tif]
