# Supplementary material for: The Germinal Center Kinase TNIK Is Required for Canonical NF-κB and JNK Signaling in B-Cells by the EBV Oncoprotein LMP1 and the CD40 Receptor
Source: PLoS Biol. 2012 Aug 14;10(8):e1001376. doi: 10.1371/journal.pbio.1001376 (PMC3419181; doi:10.1371/journal.pbio.1001376)
Supplement: Table S1 — TNIK peptides identified by mass spectrometry in the TEV eluate of HA-LMP1-liTEV-CT immunoprecipitated from LCL-TEV.5 cells. (DOC) [file pbio.1001376.s005.doc]

Table S1. TNIK Peptides detected in the TEV eluate of immunoprecipitated HA-LMP1-liTEV-CT

| **Peptide** | **Calculated Mass [Da]** | **Observed Mass [Da]** | **Sequence** | **Position in TNIK** | **Ion Score** |
| --- | --- | --- | --- | --- | --- |
| 1 | 829,5294 | 829,5334 | ALFLIPR | 241-247 | 18 |
| 2 | 1159,6205 | 1159,6384 | AIDEDLTALAK | 799-809 | 13 |
