# Supplementary material for: The Germinal Center Kinase TNIK Is Required for Canonical NF-κB and JNK Signaling in B-Cells by the EBV Oncoprotein LMP1 and the CD40 Receptor
Source: PLoS Biol. 2012 Aug 14;10(8):e1001376. doi: 10.1371/journal.pbio.1001376 (PMC3419181; doi:10.1371/journal.pbio.1001376)
Supplement: Table S2 — TNIK identification by mass spectrometry. The significance threshold for Mascot search (MOWSE score p value 0.05) was 28 and corresponds to a protein score confidence interval (C.I.) of 95%. (DOC) [file pbio.1001376.s006.doc]

Table S2. TNIK identification

| **Name** | **Species** | **Accession** | **Protein MW** | **Peptides** | **Protein Score** | ^a^**Protein Score C.I.%** |
| --- | --- | --- | --- | --- | --- | --- |
| TNIK | H. sapiens | TNIK_HUMAN | 155361 | 2 | 31 | 98,04 |

a, the maximum protein score C.I. % is 100.
